# Differences in production performance, fore-digestive tract microbiota, and expression levels of nutrient transporters of Hu sheep with different feed conversion ratio

**DOI:** 10.1128/spectrum.01423-24

**Published:** 2025-04-17

**Authors:** Xiaobin Yang, Jiangbo Cheng, Dan Xu, Chong Li, Deyin Zhang, Yukun Zhang, Kai Huang, Xiaolong Li, Yuan Zhao, Liming Zhao, Quanzhong Xu, Zongwu Ma, Huibin Tian, Xiuxiu Weng, Jie Peng, Xiaoxue Zhang, Weimin Wang

**Affiliations:** ^1^State Key Laboratory of Herbage lmprovement and Grassland Agro-ecosystems, Key Laboratory of Grassland Livestock Industry Innovation, Ministry of Agriculture and Rural Affairs; Engineering Research Center of Grassland Industry, Ministry of Education, Collegeof Pastoral Agriculture Science and Technology, Lanzhou University 628746 , Lanzhou, Gansu, China; 2 College of Animal Science and Technology, Gansu Agricultural University 74661 , Lanzhou, Gansu, China; 3 College of Veterinary Medicine, Gansu Agricultural University 74661 , Lanzhou, Gansu, China; Lerner Research Institute, Cleveland, Ohio, USA

**Keywords:** feed conversion ratio, fore-digestive tract, Hu sheep, microorganism, production performance

## Abstract

Increasing strain on feed resources has led to a gradual increase in feed input costs, making it necessary to improve feed efficiency in livestock and poultry. In this study, Hu sheep were divided into two groups (high and low feed conversion ratio [FCR]) according to the FCR. Based on 16S rDNA amplicon sequencing technology to compare rumen and small intestine microbial composition, the differences and similarities of production performance, expression level of intestinal nutrient-specific carrier, digestive enzyme activity, short-chain fatty acid (SCFA) content, muscle conventional nutrient content, and blood biochemical indexes of Hu sheep in high- and low-FCR groups were investigated, and correlation analysis was conducted. The results showed that Hu sheep in the low-FCR group had higher feed efficiency, average daily gain, and less fat deposition (*P* < 0.05). The difference in rumen microbial composition between the high- and low-FCR groups was significant (*P* < 0.05). Spearman’s correlation analysis showed that FCR was significantly associated with production performance such as body weight, fat deposition, and dressing percentage (*P* < 0.05). The levels of digestive enzyme activity and nutrient transporter carrier expression in the small intestine were higher in the low-FCR group than in the high-FCR group. Therefore, FCR can be one of the important targets of concern in Hu sheep production. Combining FCR and regulating the gastrointestinal environment of Hu sheep by nutritional means can greatly improve the production performance and economic benefit of Hu sheep.

**IMPORTANCE:**

Feed costs account for a large portion of housed sheep. The purpose of comparing the performance and intestinal microbial composition of different FCR Hu sheep is to regulate the gastrointestinal microecology in production practice. This helps livestock producers choose low-FCR Hu sheep to maximize production costs, improve efficiency, and achieve the purpose of low-carbon production.

## INTRODUCTION

Sheep, as important economic animals, are widely distributed around the world, with sheep in Asia accounting for a large part of the population (1, 2). Hu sheep, as the leading breed of housed farming in China, has excellent characteristics such as fast growth and development and strong environmental adaptability (3). Mutton is widely loved by consumers, so improving the production performance of Hu sheep has become an important research objective nowadays (4). As a commonly used index in production, feed efficiency traits can reasonably access the conversion efficiency of livestock and poultry to feed. There is also considerable room for improvement. Improved animal quality, increased production efficiency, and environmental friendliness can be achieved by selecting animals with higher feed efficiency (5). Feed conversion ratio (FCR) is one of the indices used to evaluate feed efficiency; it was proposed by Crampton et al. in 1946 and is widely used to evaluate the utilization rate of animal feed (6). Genetically, the heritability of FCR is medium; it can also be used as a benchmark for improving other traits. And researchers have found a specific correlation between FCR and fat deposition-related traits (7, 8). FCR is easy to measure in animal production and is not expensive, but feed costs account for 70% of the total animal production cost (9). Therefore, it is necessary to take FCR as the main research object to investigate the similarities and differences in gastrointestinal microorganisms, digestive enzyme activities, meat quality, and production performance of high-FCR (H-FCR) and low-FCR (L-FCR) Hu sheep.

Digestion and absorption of nutrients in the animal’s body take place primarily in the pre-digestive tract. The rumen is a vital fermentation organ in ruminants. Volatile fatty acids produced in the rumen and intestine are essential mediators of microbial–host interactions (10). The small intestine consists of the duodenum, jejunum, and ileum. The mucosal surface of the small intestine is characterized by cells (e.g., intestinal epithelial cells and intestinal villi) that help to absorb nutrients (11). The majority of nonmechanical digestion in animals takes place in the digestive tract, and carbohydrates, lipids, proteins, trace elements, and water are mainly digested and absorbed in the jejunum, which plays a vital role in the digestion and absorption of nutrients (10, 12). The sheep rumen is highly adapted to the breakdown and digestion of cellulose and other complex carbohydrates (13, 14). The gastrointestinal tract is a vital barrier that protects animals from harmful substances in the outside world, as it not only absorbs nutrients necessary for animal health but also excretes substances that are harmful to the body (8, 15). The small intestine accounts for more than 20% of the body’s oxygen consumption, making it an organ with a high metabolic rate and a great ability to adapt its function, size, and shape to the physiological needs of ruminants (11, 16). It has been found that the high and low feed efficiency of the host is significantly correlated with sequence variation in several bacterial genera (17). Whereas *Prevotella* was the microorganisms significantly enriched in the high and low feed efficiency groups, lactic acid bacteria and others were enriched in the higher feed efficiency group (18). Short-chain fatty acid (SCFA) is a key energy substance in ruminant digestion and plays a vital role in ruminant health and development (19, 20). The addition of volatile fatty acids to the feed promotes calf growth, digestion, and absorption of nutrients (21). Previous studies have shown that FCR is closely related to fat deposition, that is, animals with higher feed efficiency have less fat deposition (7, 22). Although the microbiological composition of the gastrointestinal tract of sheep with different FCR has been investigated, there is still a lack of comprehensive research on the relationship between FCR and sheep performance, as well as on the activity of digestive enzymes, expression levels of intestinal transfer vectors, muscle quality, and biochemical indexes in the intestinal tract of sheep with different FCR. Therefore, we believe that it is of great significance to carry out such research, which not only helps to improve the efficiency of feed utilization but also promotes the realization of energy saving, emission reduction, and green production. We speculate that sheep with higher feed efficiency have a richer and more diverse intestinal microbiota, a higher number and efficiency of nutrient transporters in the intestine, and greater digestive enzyme activity, allowing them to absorb nutrients more effectively. Therefore, the purpose of this study was to compare the production performance and intestinal microbial composition of different FCR Hu sheep in detail and to clarify the dominant position of low-FCR Hu sheep in production. By using FCR as the selection index, the early selection of sheep production can maximize production efficiency.

## MATERIALS AND METHODS

### Animals and groups

The 310 male Hu sheep were selected from the following four farms: Changxing Yongsheng Livestock Co., Ltd. (Huzhou, Zhejiang Province), Zhejiang Sino Ecological Agriculture Co., Ltd. (Hangzhou, Zhejiang Province), Gansu Runmu Biotechnology Co., Ltd. (Jinchang, Gansu Province), and Hangzhou Pangda Agricultural Development Co., Ltd. (Hangzhou, Zhejiang Province). All the test sheep were weaned at 56 days of age and then transferred to DeFu Agricultural Technology Co., Ltd., in Minqin County for breeding; each sheep has its own stall and is kept in its own pen (0.8m × 1.0m) indoor. After a transition period of 14 days and a pre-feeding period of 10 days, the sheep were allowed to eat and drink water freely, and the feeding management was consistent throughout the test period. All sheep were fed individually. At the beginning of the formal test period, at 80 days of age, body weight was measured with a calibrated electronic scale, and body size traits such as body height, body length, chest circumference, and cannon circumference were measured with a measuring tape before morning feeding, until 180 days of age. The total mixed pellet feed formula of this experiment was designed according to the “Feeding Standard for Sheep and Goats for Meat (NY/T 816–2004),” and the pellet feed was processed by Gansu Runmu Biological Engineering Co., Ltd. (Jinchang, Gansu Province), according to the formula. The feed formula is shown in Table S1.

The feed intake of each sheep was recorded every 10 days as the difference between offered and refused feed, and the average daily feed intake (ADFI) was calculated. According to the FCR classification of 80–180d, the FCR calculation method is ADFI/average daily gain (ADG) (23). After screening out outliers by the mean ± 3 × standard deviation, according to the proportion of 5%, a total of 30 sheep were selected from all the sheep as H-FCR group and L-FCR group, with 15 sheep in each group.

### Sample collection and processing

At 180 days of age, all experimental sheep were slaughtered. Before slaughter, 4 to 5 mL of blood was collected from the jugular vein of 30 Hu sheep in the H-FCR and L-FCR groups for the determination of blood biochemical indexes. All sheep were fasted for 12 hours before slaughter. In sheep rearing and trait determination, we fixed one to two persons as much as possible; all the sheep can move freely in the pen, feed, drink, rest, etc. during the rearing process; and there was a blower to cool down the temperature when the weather is hot. At the same time, we will clean up the feces in the pen in time to ensure the environmental hygiene. All sheep are slaughtered under the supervision and guidance of a professional veterinarian, where they are first knocked unconscious and then euthanized by bloodletting through the jugular vein, to ensure the animal welfare of experimental sheep as much as possible. The rumen and intestine were also ligated after slaughter to avoid contamination due to the movement of contents. Samples of rumen and small intestinal contents, small intestinal mucosa, small intestinal tissue, and longest dorsal muscle were collected. Adipose tissue (tail fat, mesenteric fat, and perirenal fat) was collected with reference to Zhang et al. (24). The longest back muscle samples were de-acidified for meat quality determination (fat, moisture, salt, protein, and collagen) using a meat quality tester (Foodscan 2, Foss China, China, Beijing), and three biological replicates were made for each meat sample, with two technical replicates for each biological replicate. The collection method of the mucosa was based on the research of Wang et al. (25). Samples were immediately frozen in liquid nitrogen and then subsequently transferred to a laboratory −80°C ultra-low temperature freezer for storage. Alpha-amylase, lipase, and trypsin were determined according to the instructions of the corresponding assay kits (Nanjing Jiancheng Bioengineering Institute, China). The contents of the rumen and small intestine were sequenced by Beijing Nuohe Zhiyuan Technology Co., Ltd. (Beijing, China) for 16S rDNA sequencing. The blood samples were then subsequently centrifuged, and the serum was aspirated and stored in a refrigerator at −80°C for the determination of blood biochemical parameters (including alanine aminotransferase, aspartate aminotransferase, total bilirubin, direct bilirubin, total protein, albumin, alkaline phosphatase, creatinine, triglyceride, lactate dehydrogenase, creatine kinase, and glucose). Blood biochemistry was measured using a Biochemical Analyzer XL 640 (Erba, Germany) with the addition of kits for the corresponding biochemical parameters (Medicalsystem Biotechnology Co., Ltd., China). The SCFA content in the rumen and small intestine was measured by a Pano gas chromatograph (A91 PLUS, Pano Instruments Co., Ltd., China); the column was a S.N17-11-010 capillary column (Analytical Technology, China); the chromatographic conditions were 250°C in the injection port, 5.4 mL/min nitrogen flow rate, a split ratio of 5:1, an injection volume of 1 µL, and a programmed heating mode (190°C for 3 min, then 30°C/min to 240°C, hold for 1 min), flame ion detector (FID) 250°C, and FID air, hydrogen, and nitrogen flow rates of 300, 30, and 20 m L/min, respectively.

### Total DNA extraction and PCR amplification

Total genomic DNA was extracted from the samples using the cetyltrimethylammonium bromide method. DNA concentration and purity were monitored on 1% agarose gel. Depending on the concentration, DNA was diluted to 1 μg/μL using sterile water and stored at −20°C.

Genomic DNA extracted from small intestine samples was used as a template to amplify the 16S V3-V4 regions using the primer set 341 F-806R (forward primer F: CCTAYGGGRBGCASCAG and reverse primer R: GGACTACNNGGGTATCTAAT). All PCR reactions were performed with 15-µL Phusion High-Fidelity PCR Master Mix (New England Biolabs); 0.2-µM forward and reverse primers and approximately 10-ng template DNA. Thermal cycling consisted of an initial denaturation at 98°C for 1 min, followed by 30 cycles of denaturation at 98°C for 10 s, annealing at 50°C for 30 s, and extension at 72°C for 30 s. Finally, 72°C for 5 min.

### Library preparation and sequencing

Sequencing libraries were generated using TruSeq DNA PCR-Free Sample Preparation Kit (Illumina, USA) according to the manufacturer’s recommendations, and index codes were added. Library quality was assessed using Qubit 2.0 Fluorometer (Thermo Scientific) and Agilent Bioanalyzer 2100 system. At last, the library was sequenced on an Illumina NovaSeq platform, and 250-bp paired-end reads were generated.

### Sequencing data analysis and processing

After sequencing, the raw data for each sample were obtained. Paired-end reads were assigned to samples based on their unique barcode and truncated by cutting off the barcode and primer sequence. Paired-end reads were merged using FLASH (VI.2.7, http://ccb.jhu.edu/software/FLASH/) (26), which was designed to merge paired-end reads when at least some of the reads overlap the read generated from the opposite end of the same DNA fragment, and the splicing sequences were called raw tags. Quality filtering on the raw tags was performed under specific filtering conditions to obtain the high-quality clean tag (27) according to the FASTP. The tags were compared with the reference database (Silva database, https://www.arb-silva.de/) using the UCHIME algorithm (http://www.drive5.com/usearch/manual/uchime_algo.html) (28) to detect chimera sequences, and then the chimera sequences were removed (29). Then the effective tags were finally obtained. The sequences were clustered into amplicon sequence variant feature sequences according to 100% similarity. To ensure that the sequencing depth met the analysis needs, we constructed sparse curves. BLAST algorithm was used to compare and annotate the sample sequence with the reference sequence (SILVA138 database) to determine the community composition at each species level (kingdom, phylum, class, order, family, genus, and species).

The calculation of alpha and beta diversity is based on QIIME 2 (version 2023.5.1) and R (version 4.2.0) software. We calculate three metrics: the Chao1, Shannon, and Simpson indices. QIIME two calculated unweighted UniFrac distances and performed principal coordinate analysis (PCoA) and unweighted pair-group method with arithmetic means (UPGMA) clustering in R. The linear discriminant analysis effect size (LEfSe) analyses used the microbiome marker package in R software. The relationship between microorganisms and traits was examined using Spearman correlation analysis. The correlation analysis used the psych, pheatmap, and ggplot2 packages in R, with *P* < 0.05 used as the significance criterion.

### Tissue expression analysis

Six Hu sheep were randomly selected from each H-FCR and L-FCR groups for tissue expression analysis. These genes were *SGLT1* (sodium-dependent glucose transporter 1), *GLUT2* (glucose transporter 2), *GLUT5* (glucose transporter 5), *PEPT1* (solute carrier family 15 member 1 [SLC15A1], peptide transporter 1), *CAT1* (SLC7A1, solute carrier family 7 member 1), *rBAT* (SLC3A1, solute carrier family 3 member 1), *B^0^AT* (SLC6A19, solute carrier family 6 member 19), *EAAT3* (SLC1A1, solute carrier family 1 member 1), and *FATP4* (SLC27A4, solute carrier family 27 member 4). Specific quantitative reverse transcription PCR (qRT-PCR) primers for tissue expression analysis are listed in Table S2. For each gene, based on the recommended temperatures of the primer manufacturers, we set a gradient temperature to screen the optimal temperature for each gene, and we optimized qPCR conditions for gene quantification based on these temperatures. Reverse transcription of RNA into cDNA was performed according to the kit instructions (Transgen, China). The BIO-RAD CFX96 Touch Real-time PCR Detection System (BIO-RAD, USA) was used for qPCR detection. The reaction system for qRT-PCR was performed according to the study by Wang et al. (30), and data analysis was performed using the 2^−ΔΔCt^ method with *GAPDH* (glyceraldehyde 3-phosphate dehydrogenase) as the reference gene (31).

### Statistical analysis

All traits data from the experimental Hu sheep were summarized and calculated using Excel 2021; phenotypic differences between groups and Spearman correlation analyses were performed using SPSS 23.0 software. Differences in traits (growth traits, feed intake, slaughter traits, digestive enzyme activities, meat quality, blood biochemicals, etc.) between the H-FCR and L-FCR groups were analyzed using *t*-test. One-way analysis of variance (ANOVA) was used to compare differences in relative abundance between rumen and small intestine phyla. In statistical tests for alpha and beta diversity analyses, different groups were compared using *t*-test and rank sum tests, which were corrected for multiple comparisons. Differences were considered significant when *P* < 0.05.

## RESULTS

### Correlation analysis of FCR with production performance

**Fig 1:**
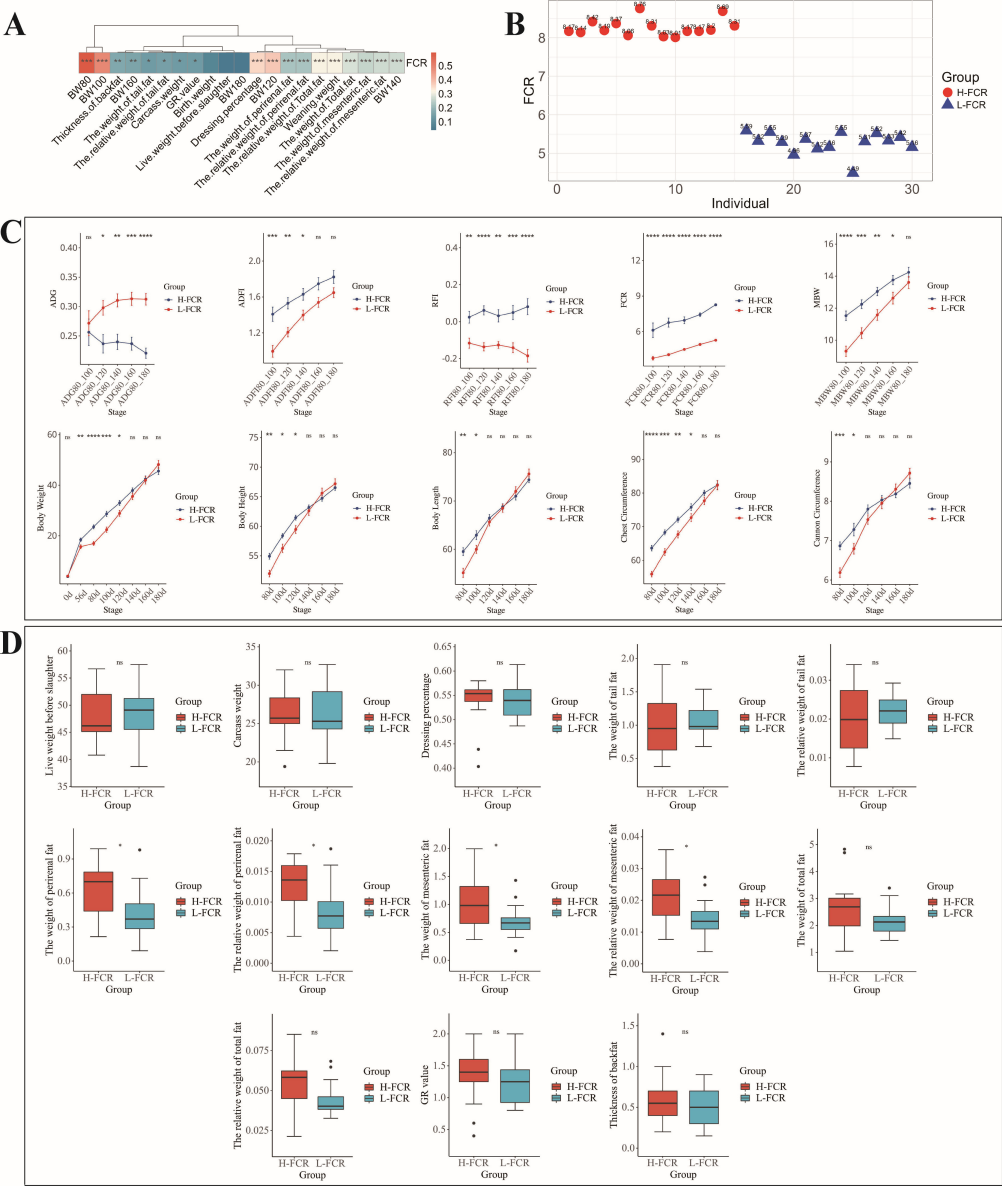
FCR and production performance comparison. (A) Spearman correlation heat map of FCR and production performance. (B) Scatter plot of FCR values of individual Hu sheep in H-FCR and L-FCR groups. (C) Comparison of feed efficiency and related traits of body weight and body size between H-FCR and L-FCR groups. (D) Comparison of slaughter performance of Hu sheep in H-FCR and L-FCR groups. * or ** or *** or **** indicates a significant difference (*P* < 0.05); ns shows no significant difference (*P* > 0.05).

To investigate the correlation between FCR and production performance, Spearman’s correlation analysis was performed based on production data from 310 Hu sheep. The results are shown in Fig. 1A and indicate that FCR was significantly correlated with the (relative) weight of tail fat; perirenal fat; mesenteric fat; total fat; carcass fat content (GR) value; thickness of backfat; weaning weight; body weight at 80, 100, 120, 140, and 160 days; carcass weight; and dressing percentage (*P* < 0.05). This suggests a strong need to use FCR as an indicator for selective retention of Hu sheep.

### Comparative study of production performance

#### Comparison of feed efficiency-related traits and growth traits

In order to explore the differences in traits related to feed efficiency and growth traits of Hu sheep with different FCR, we compared ADG, ADFI, residual feed intake (RFI), FCR, mid-test metabolic body weight (MBW), and growth traits at different stages. In Fig. 1B, the individual FCR of sheep in the H-FCR and L-FCR groups are presented. The results showed that the ADG, ADFI, RFI, FCR, and MBW of the L-FCR group at different stages were significantly lower than those of the H-FCR group (*P* < 0.05). Significant differences were observed in body weight at 56, 80, 100, and 120 days between the H-FCR and L-FCR groups (*P* < 0.05) (Fig. 1C).

#### Comparison of slaughter performance and fat deposition traits

The results showed that there were significant differences in the (relative) weight of perirenal fat and mesenteric fat between the H-FCR and L-FCR groups (*P* < 0.05). There were no significant differences in the other traits (*P* > 0.05) (Fig. 1D).

### Sequencing data overview

The contents of the rumen, duodenum, jejunum, and ileum of a total of 30 Hu sheep in the H-FCR and L-FCR groups were sequenced for 16S rDNA. The statistical results of the sequencing data are shown in Table S3. A total of 110 samples (28 samples from duodenum, 30 from jejunum, 22 from ileum, and 30 from rumen) were sequenced due to low content or the samples themselves, and a total of 8,817,153 raw reads were obtained. After splicing, 8,433,296 raw tags were obtained, resulting in 8,367,264 clean tags after filtering out low-quality and short sequences. Finally, 6,905,950 effective tags were used for analysis. The average length of each sequence was 415.5 bp. To confirm the suitability of the sequencing results for our research, we performed an observed features curve analysis of the samples. Fig. S1A showed that as the sequencing depth increases, the curve gradually flattens, indicating that the sequencing results are suitable for analysis.

### Species diversity analysis

To explore the microbial community composition of the rumen and small intestine in the H-FCR and L-FCR groups of Hu sheep, we performed alpha diversity analysis (Fig. S1B). There were no significant differences in the Shannon index, Simpson index, and Chao1 index of the duodenum, jejunum, ileum, and rumen between the two groups (*P* > 0.05). We also analyzed the microbial alpha diversity of these four positions in the rumen and small intestine (Fig. S1C). The results showed that the rumen and duodenum, jejunum, and ileum showed significant differences in alpha diversity, which was higher in the rumen than that in the small intestine.

The PCoA results based on the Bray–Curtis distances showed overlap between the H-FCR and L-FCR groups in different positions, but there was a significant difference in the rumen microbial composition between the H-FCR and L-FCR groups of Hu sheep (Fig. S1D). The results of beta diversity analysis of rumen, duodenum, jejunum, and ileum are shown in Fig. S1E, which indicated that there was a significant separation between rumen and duodenum, jejunum, and ileum (*P* < 0.05). There were no significant differences in the microbiological composition of the different segments of the small intestine.

### Species composition analysis of the rumen and small intestine

In order to explore the species composition of the rumen and small intestine at the phylum level, we plotted bar graphs of the top 10 phyla in relative abundance (Fig. S1F and G). The results showed that the relative abundance of *Bacteroidota* and *Firmicutes* in the rumen and *Proteobacteria* and *Firmicutes* in the small intestine was the highest. At once, we immediately performed an ANOVA of the top 10 phyla in terms of relative abundance at the phylum level and found that *Euryarchaeota*, *Actinobacteriota*, *Bacteroidota*, *Campilobacterota*, *Desulfobacterota*, *Fibrobacterota*, *Firmicutes*, *Patescibacteria*, *Proteobacteria*, *Spirochaetota*, and *Verrucomicrobiota* differed significantly in relative abundance in the rumen and small intestine of Hu sheep in the H-FCR and L-FCR groups (Table 1). In contrast, the relative abundance of *Desulfobacterota* in the duodenum, *Campilobacterota* in the ileum, and *Spirochaetota* in the rumen between the H-FCR and L-FCR groups was significant (*P* < 0.05).

**TABLE 1:** Descriptive statistics of relative abundance of top 10 phyla in the rumen and small intestine H-FCR and L-FCR groups^*a*^

Taxonomic name	Duodenum				Jejunum				Ileum				Rumen			
	H-FCR		L-FCR		H-FCR		L-FCR		H-FCR		L-FCR		H-FCR		L-FCR	
	Mean	SE	Mean	SE	Mean	SE	Mean	SE	Mean	SE	Mean	SE	Mean	SE	Mean	SE
Euryarchaeota	0.03^bc^	0.01	0.06^b^	0.02	0.13^a^	0.02	0.11^a^	0.03	0.06^b^	0.02	0.03^bc^	0.01	0.02^bc^	0.01	0.01^c^	0.00
Actinobacteriota	0.04^cd^	0.01	0.13^abc^	0.04	0.15^ab^	0.04	0.21^a^	0.05	0.10^bcd^	0.04	0.06^bcd^	0.02	0.01^d^	0.00	0.01^d^	0.00
Bacteroidota	0.04^bc^	0.02	0.08^bc^	0.03	0.01^c^	0.00	0.01^c^	0.00	0.07^bc^	0.04	0.11^b^	0.03	0.46^a^	0.02	0.48^a^	0.03
Campilobacterota	0.00^b^	0.00	0.00^b^	0.00	0.00^b^	0.00	0.00^b^	0.00	0.00^b^	0.00	0.00^a^	0.00	0.00^b^	0.00	0.00^b^	0.00
Desulfobacterota	0.00^b^	0.00	0.01^a^	0.01	0.00^b^	0.00	0.00^b^	0.00	0.01^ab^	0.01	0.01^ab^	0.00	0.00^b^	0.00	0.00^b^	0.00
Fibrobacterota	0.01^b^	0.01	0.00^b^	0.00	0.00^b^	0.00	0.00^b^	0.00	0.00^b^	0.00	0.01^b^	0.01	0.03^a^	0.01	0.04^a^	0.01
Firmicutes	0.27^b^	0.06	0.29^b^	0.04	0.31^b^	0.04	0.27^b^	0.04	0.65^a^	0.06	0.65^a^	0.04	0.32^b^	0.03	0.33^b^	0.02
Patescibacteria	0.01^c^	0.00	0.02^abc^	0.01	0.03^ab^	0.01	0.02^abc^	0.01	0.04^a^	0.02	0.02^abc^	0.01	0.01^bc^	0.00	0.01^bc^	0.00
Proteobacteria	0.59^a^	0.08	0.40^b^	0.10	0.36^b^	0.07	0.38^b^	0.08	0.07^c^	0.02	0.10^c^	0.03	0.04^c^	0.01	0.07^c^	0.03
Spirochaetota	0.01^c^	0.01	0.00^c^	0.00	0.00^c^	0.00	0.00^c^	0.00	0.00^c^	0.00	0.00^c^	0.00	0.11^a^	0.02	0.06^b^	0.01
Verrucomicrobiota	0.00^b^	0.00	0.00^b^	0.00	0.00^b^	0.00	0.00^b^	0.00	0.01^a^	0.00	0.01^a^	0.00	0.00^b^	0.00	0.00^b^	0.00

^*a*^ Different lowercase letters in the same row of shoulder marks indicate significant differences.

The Venn diagram showed the biomarkers from the rumen, jejunum, and ileum and found that there were no common biomarkers among these three groups (Fig. S1H). We performed LEfSe analysis on the microbiota of the rumen, duodenum, jejunum, and ileum in H-FCR and L-FCR Hu sheep (Fig. S2). The results showed that microbial biomarkers were statistically significant in different groups. The rumen had the greatest number of differentially abundant microbiota. *Clostridia*, *Hungateiclostridiaceae*, *Saccharofermentans*, and *Aeromonadales* are the biomarkers of the H-FCR of the jejunum. *Methanobrevibacter_millerae*, *Burkholderiaceae*, *Ralstonia*, and *Burkholderiales* are the biomarkers of the H-FCR of the ileum. *Bacteroidaceae*, *Bacteroides*, and *Roseburia* are the biomarkers of the L-FCR of the ileum.

**Fig 2:**
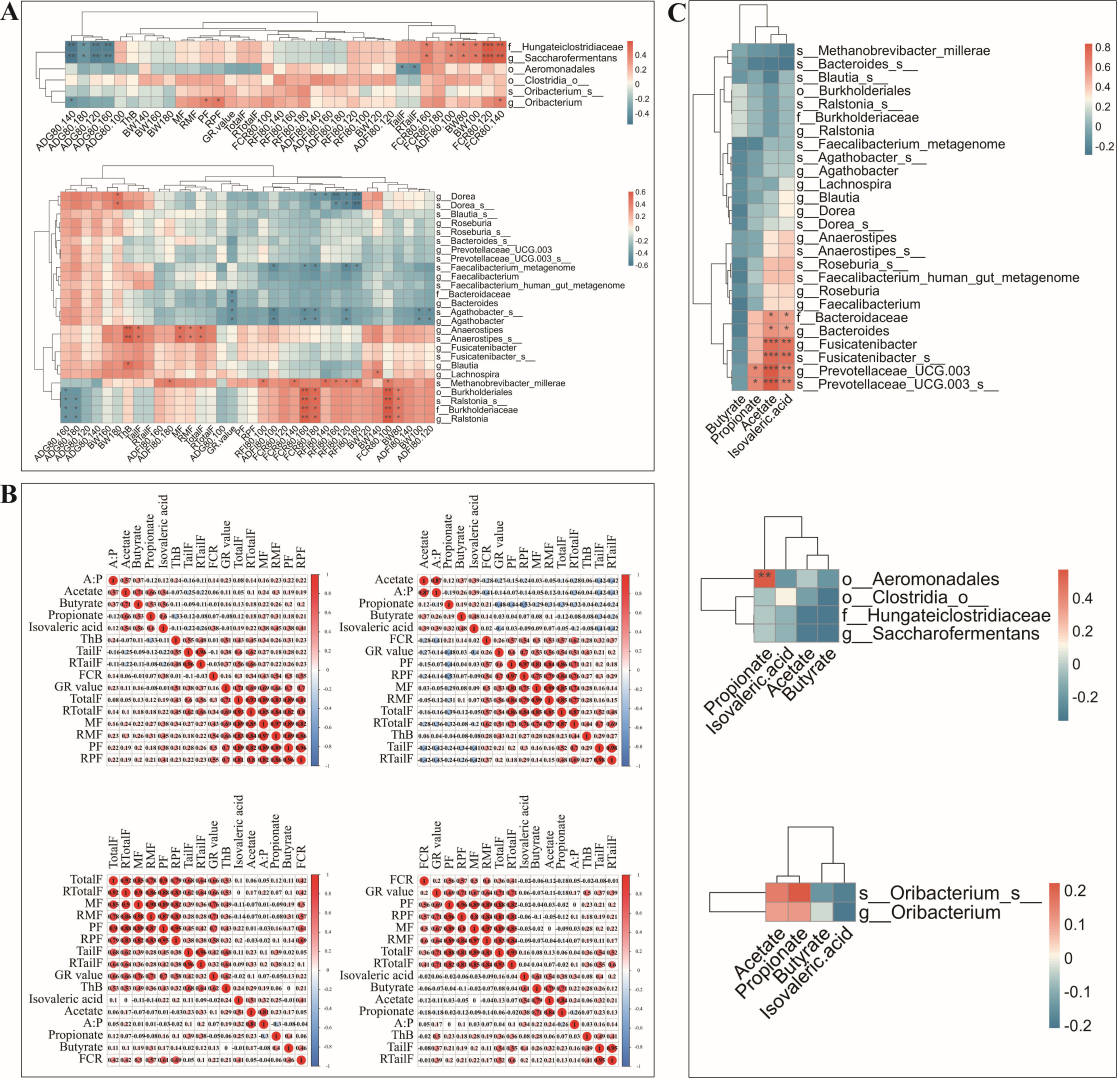
Spearman correlation analysis. (A) Correlation heat map of biomarkers of the rumen, jejunum, and ileum with body weight, feed efficiency, and fat deposition traits. Abbreviations are defined as follows: ThB: backfat thickness, TailF: tail fat weight, RTailF: relative tail fat weight, MF: mesenteric fat weight, RMF: relative mesenteric fat weight, TotalF: total fat weight, RTotalF: total fat relative weight, PF: perirenal fat weight, RPF: relative perirenal fat weight. The same as below. (B) Correlation analysis between SCFA of the duodenum, jejunum, ileum, and rumen and production performance. A:P: ratio of acetate to propionic acid. (C) Correlation analysis of biomarkers and VFA in the leum, jejunum, and rumen. * or ** or *** indicates a significant difference (*P* < 0.05).

We performed a correlation analysis of the six biomarkers in the rumen and jejunum, along with 26 biomarkers in the ileum of Hu sheep, focusing on body weight, feed efficiency, and fat deposition traits (Fig. 2A). The results indicated a correlation between Hu sheep’s biomarkers from the rumen, jejunum, and ileum and the body weight, feed efficiency, and fat deposition traits. These key biomarkers included Hungateiclostridiaceae, *Saccharofermentans*, Aeromonadales, *Oribacterium*, *Dorea*, *Agathobacter*, *Faecalibacterium_metagenome*, Bacteroidaceae, *Anaerostipes*, *Blautia*, *Lachnospira*, *Methanobrevibacter_millerae*, *Burkholderiales*, and *Ralstonia*. The results of the correlation analysis of jejunum and rumen biomarkers indicated that *Burkholderiaceae* and *Saccharofermentans* were significantly positively correlated with FCR and early body weight and significantly negatively correlated with ADG. *Aeromonadales* was significantly negatively correlated with tail fat weight, while *Oribacterium* was significantly positively correlated with perirenal fat weight and FCR and significantly negatively correlated with ADG. The results of the correlation analysis of ileum biomarkers indicated that *Anaerostipes* was significantly positively correlated with fat deposition traits. *Methanobrevibacter_millerae*, *Burkholderiales*, *Ralstonia*, and *Burkholderiaceae* were significantly positively correlated with FCR and significantly negatively correlated with ADG.

### SCFAs in the rumen and small intestine

**Fig 3:**
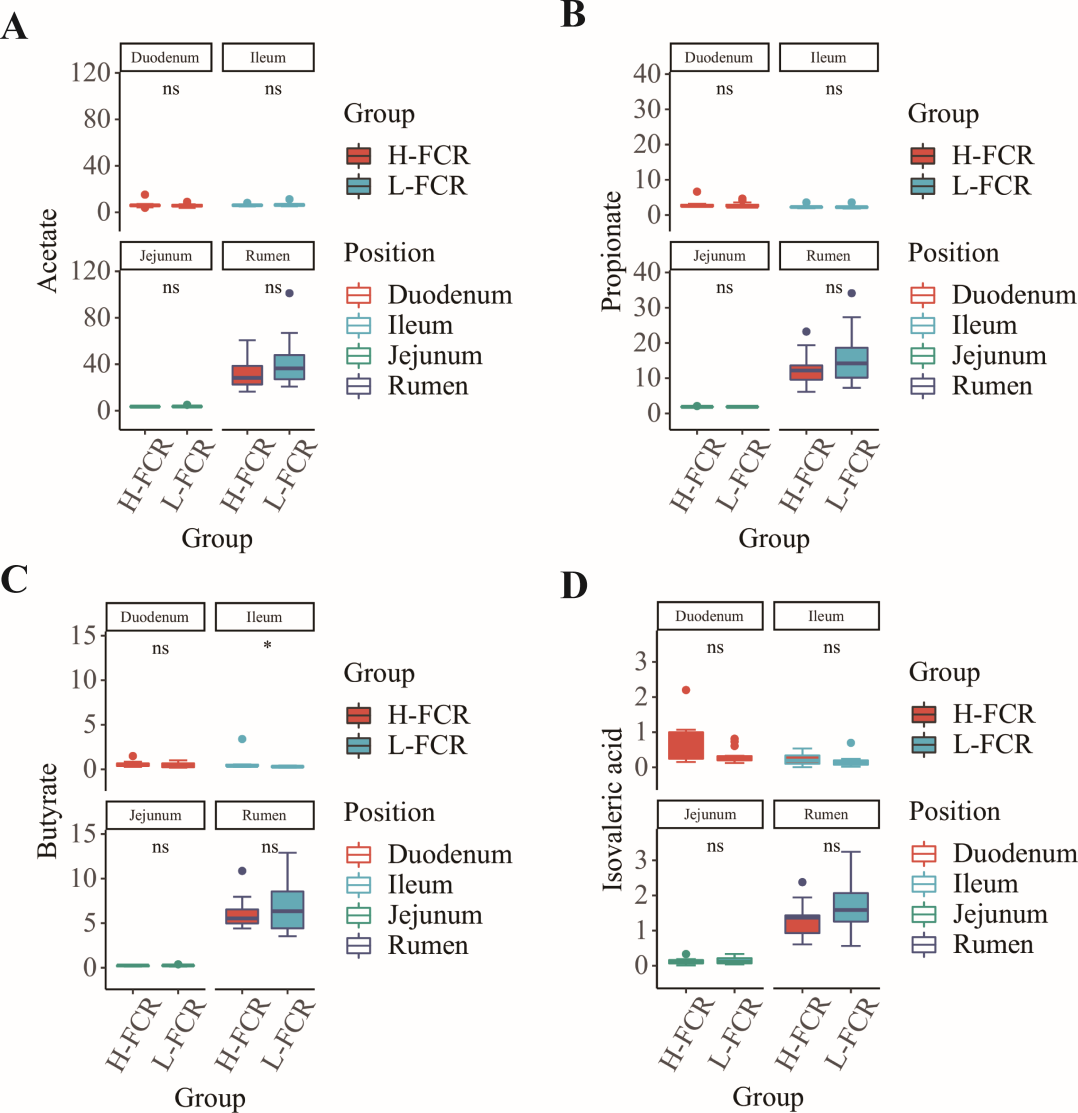
Box plot of rumen and small intestine SCFA content. Box plot of SCFA content in H-FCR and L-FCR groups of Rumen and small intestine (A–D are acetate, propionate, butyrate, and isovaleric acids, respectively). * indicates a significant difference (*P* < 0.05), and ns shows no significant difference (*P* > 0.05).

Correlation analysis was performed to further clarify the relationship between SCFA content in the rumen and small intestine with FCR and fat deposition traits (Fig. 2B). The results showed that SCFAs in rumen and small intestine were moderately or weakly correlated with FCR and fat deposition traits. To further understand the relationship between the relative abundance of markers and SCFAs, we then correlated SCFAs with the relative abundance of biomarkers (Fig. 2C). The results showed that *Aeromonadales* in the jejunum were significantly correlated with the amount of propionate; *Bacteroidaceae*, *Bacteroides*, and *Fusicatenibacter* in the ileum were significantly correlated with the amount of acetate and isovaleric acid, and *Prevotellaceae_UCG_003* was significantly correlated with acetate, propionate, and isovaleric acid. Rumen biomarkers were correlated with SCFA content but were not statistically significant. We also compared the content of SCFAs in the rumen, duodenum, jejunum, and ileum of H-FCR and L-FCR groups (Fig. 3). The results showed a significant difference in butyric acid content in the ileum between the H-FCR and L-FCR groups, while the other SCFAs did not show significant differences across various sites in the different FCR groups.

### RNA expression levels in the small intestine

**Fig 4:**
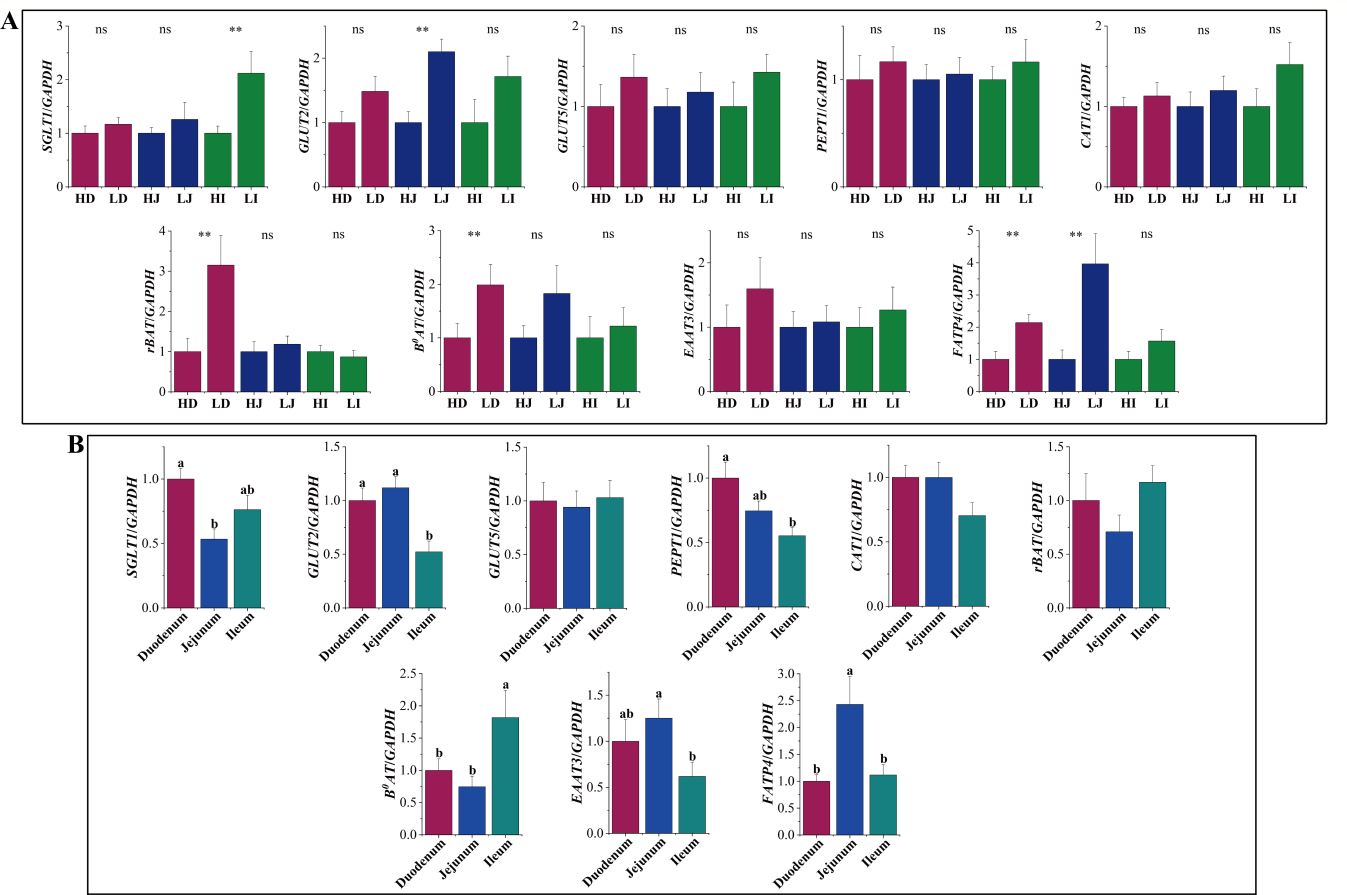
Expression levels of nutrient transporter carriers. (A) Expression levels of nutrient transporter carriers in the small intestine of H-FCR and L-FCR groups. ** means a significant difference (*P* < 0.05), and ns means no significant difference (*P* > 0.05). (B) Expression levels of nutrient transfer vectors in the small intestine. Different lowercase letters indicate significant differences (*P* < 0.05).

To investigate differences in the digestion and absorption of conventional nutrients in the small intestine of Hu sheep between the H-FCR and L-FCR groups, we performed tissue expression analyses of sugar transporter proteins, amino acid transporter proteins, and fatty acid transporter proteins (Fig. 4A). The results showed significant differences in the expression levels of *rBAT*, *B^0^AT*, and *FATP4* in the duodenum, *GLUT2* and *FATP4* in the jejunum, and *SGLT1* in the ileum were significantly different between the H-FCR and L-FCR groups. (*P* < 0.05). However, there were no significant differences in the expression levels of the remaining genes in the small intestine of the H-FCR and L-FCR groups (*P* > 0.05).

In addition, to verify the differences in nutrient transport capacity in different parts of the small intestine, we compared the expression levels of these transporter protein vectors in different parts of the small intestine (Fig. 4B). It was found that the expression levels of *SGLT1*, *GLUT2*, and *PEPT1* were higher in the duodenum, while *EAAT3* and *FATP4* were higher in the jejunum, and *B^0^AT* was higher in the ileum (*P* < 0.05).

### Digestive enzyme activity of the small intestinal mucosa

**Fig 5:**
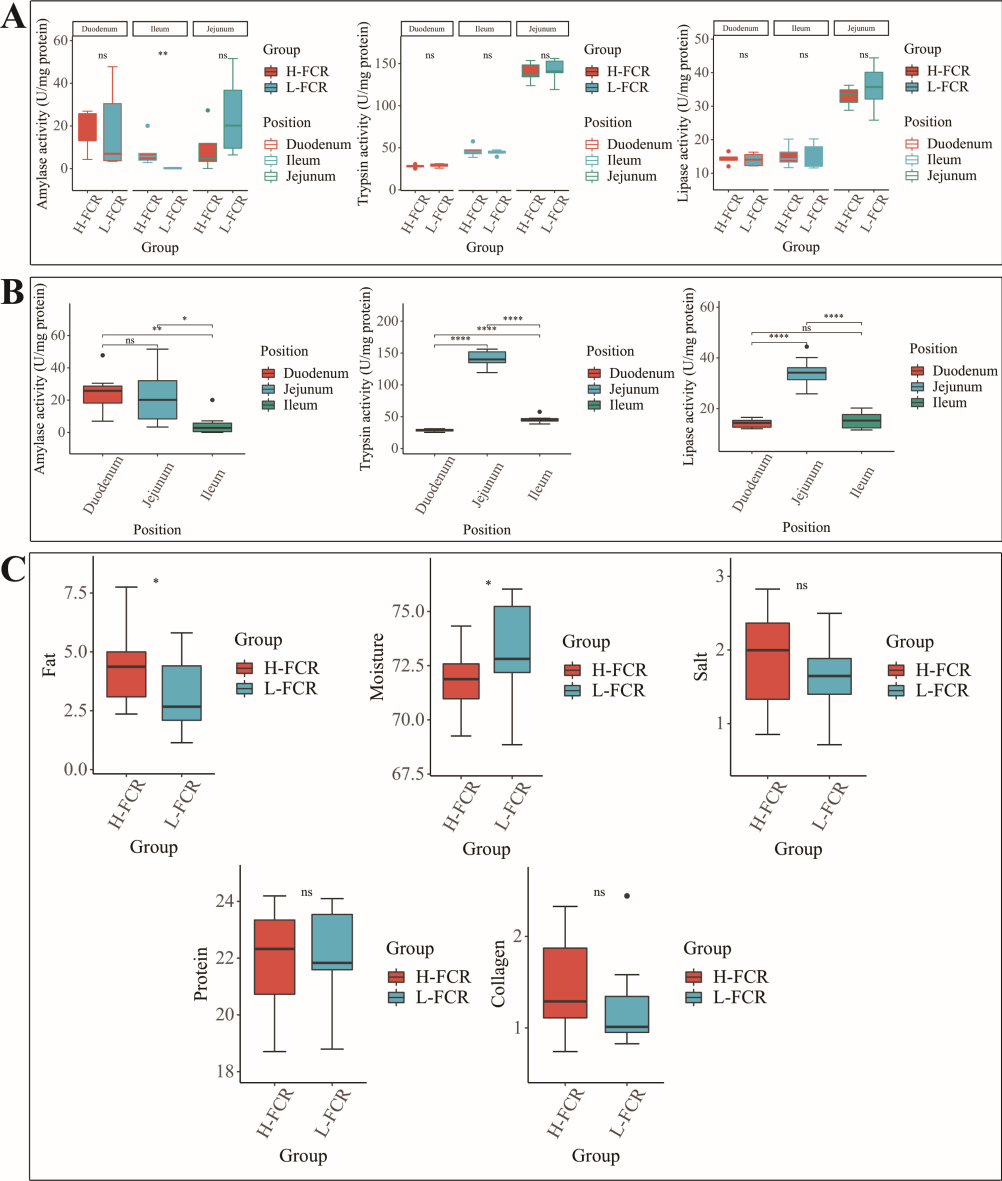
Box plot of digestive enzyme activities in the small intestine and mutton quality in H-FCR and L-FCR groups. (A) Activity of amylase, trypsin, and lipase in the intestinal mucosa in H-FCR and L-FCR groups. (B) Digestive enzyme activities of the duodenum, jejunum, and ileum. (C) H-FCR and L-FCR group Hu sheep mutton quality boxplot. *, **, or **** means a significant difference (*P* < 0.05), and ns indicates no significant difference (*P* > 0.05).

In order to compare the ability of the small intestine to digest and absorb food, we measured the enzyme activities of the small intestinal mucosa of Hu sheep in different FCR groups (Fig. 5A). The results showed that amylase activity in the ileum significantly differed between the H-FCR and L-FCR groups (*P* < 0.05), while the other enzymes did not show significant differences (*P* > 0.05). In addition, we found that the activity of digestive enzymes in the jejunum was significantly higher than that in the duodenum and ileum (Fig. 5B) (*P* < 0.05).

### Mutton quality determination and blood biochemical indicators

To further investigate whether there was a difference in meat quality between the H-FCR and L-FCR groups of Hu sheep, we carried out measurements of the routine nutrient content of the mutton (Fig. 5C). The indices measured included fat, moisture, salt, protein, and collagen. The results showed that the muscle fat content of Hu sheep in the L-FCR group was significantly lower than that in the H-FCR group, while the moisture content showed an opposite trend (*P* < 0.05), and the other meat quality indices did not show significant differences (*P* > 0.05).

To compare the organ function, metabolism, and nutritional status of Hu sheep in the H-FCR and L-FCR groups, we measured blood biochemical indices (Table S4). The results indicated that there were no significant differences in the blood biochemical indicators between the two groups (*P* > 0.05).

## DISCUSSION

### FCR and production performance

FCR has been widely employed as an indicator for evaluating the efficiency of livestock and poultry feed utilization. The effective selection of feed efficiency significantly contributes to enhancing the sustainability and economic viability of ruminant production (32). Feed costs constitute more than two-thirds of the total production costs in housed sheep farming (9). Therefore, breeding sheep with high feed efficiency, rapid growth, and minimal fat deposition is crucial for enhancing the economic benefits of sheep farming and meeting consumer demands. This approach not only helps reduce production costs but also contributes to the development of environmentally friendly and low-carbon animal husbandry.

In the present study, FCR was significantly associated with fat content, dressing percentage, and body weight in Hu sheep. Notably, the muscle fat content of Hu sheep in the L-FCR group was lower. It has been shown that FCR is highly correlated with most production traits, and using FCR as a selection metric does not negatively impact meat production performance. Additionally, FCR is a trait with medium heritability (33). In contrast, selecting animals with low RFI may lead to an increase in carcass fat (34). Detweiler et al. (35) found significant differences in slaughter weight and hot carcass weight among Angus cattle with different ADG but no differences in dressing percentage between high- and low-ADG groups. Zhao et al. (36) demonstrated that fat deposition and feed efficiency traits were positively correlated. Danielle et al. found that Santa Ines sheep in the high-RFI efficiency group fed less than those in the low-RFI efficiency group (37); the results of this manuscript are similar to this study. Zeng et al. found that there were no differences in body weight and ADG between the high- and low-RFI groups; there were no differences in biochemical indices between the two groups except for glucose. In the present study, the L-FCR group had better production performance than the H-FCR group, and there was no difference in serum indices, from which we hypothesized that the differences might be due to the different ages and group sizes of the Hu sheep (38). This suggests that L-FCR Hu sheep exhibit better feed efficiency and growth rates, and breeding sheep with L-FCR can reduce feed input costs and minimize fat deposition.

### Microbiota composition, VFA content, and FCR

The relationship between gut microbes and the host encompasses nutrient metabolism, immune regulation, digestion, absorption, and protection, which are critical for host health and immune function (39, 40). In this study, we found correlations between FCR, fat deposition, and SCFA. SCFAs, such as acetic, propionic, and butyric acids, are crucial for fat synthesis and metabolism, providing about 70% of the host’s energy (41, 42). Microorganisms have been shown to regulate fat deposition in the body (43). Wang et al. (44) found that the gut microbial communities of the three breeds of experimental sheep were more focused on fatty acid and bile acid synthesis. This suggests that animals with different phenotypes may exhibit specific gastrointestinal microbiota at the genetic level. Tan et al.’s (45) study on the gut microbiology of pigs revealed significant differences in alpha diversity in the ileum of H-FCR and L-FCR pigs. This indicates that when selecting for FCR, the microbial diversity of the small intestine remains relatively consistent. Additionally, specific genera such as *Oribacterium* in the rumen, *Aeromonadales* in the jejunum, and *Bacteroidaceae*, *Agathobacter*, and *Anaerostipes* in the ileum have been implicated in the anabolism of body fat (46–49). *Bacteroidota* and *Firmicutes* are the main dominant phyla in the rumen, a finding that is consistent with the results of Guo et al. (50) in Tibetan goats. And *Proteobacteria* are mainly involved in the production of propionate, whereas *Fibrobacterota* contribute to the degradation of cellulose and hemicellulose. In addition, it has been shown that there is a correlation between increased propionate and reduced methane emissions in cattle (51). Zeng et al. (38) found that the L-RFI group had significantly higher propionic and acetic acid and less methane production than the H-RFI group. In the present study, acetic and propionic acids were higher but not significant in the L-FCR group than in the H-FCR group. Therefore, L-FCR sheep may have better gluconeogenesis function. We hypothesized that these specific phylum and their genera act in the gut by degrading substances such as cellulose in the host diet, which in turn affects the production of SCFA such as propionic acid and its content. These SCFAs enter the host’s liver and other tissues through the bloodstream and participate in important physiological processes such as fatty acid synthesis, energy metabolism, and glucose metabolism. In particular, propionic acid can be converted to glucose by the liver, thereby boosting the host’s energy supply, which in turn has an integrated effect on feed efficiency and other economic traits in sheep.

SCFAs serve as a crucial link between gut microorganisms and the host’s tissues, helping maintain homeostasis and overall health. Microorganisms and SCFAs work synergistically to influence body fat synthesis and metabolism (52). Notably, there is a significant negative correlation between propionic acid levels and fat deposition in the jejunum, highlighting its important role in lipid digestion and metabolism. Propionic acid acts as a precursor for gluconeogenesis, facilitating communication between the gluconeogenesis pathway and fat deposition, while promoting gluconeogenesis as its concentration in the gut increases (53). This process is associated with enhanced metabolic activity and may help mitigate fat accumulation. Specific microorganisms, such as *Agathobacter*, *Methanobrevibacter*, and *Prevotellaceae_UCG-003*, showed significant associations with feed efficiency and fat traits in both H-FCR and L-FCR groups. By regulating SCFA production and metabolism, gut microbes can influence feed utilization efficiency, impacting animal growth and health through alterations in energy and amino acid metabolism. Carmichael et al. (54) noted a higher abundance of *Methanobrevibacter* in the inefficient group of cattle, indicating energy loss as methane. Our study found that the abundance of *Methanobrevibacter* was lower in the L-FCR group and positively correlated with both FCR and RFI. An increased presence of *Euryarchaeota*, including *Methanobrevibacter*, aligns with these findings, suggesting that higher methanogenic bacteria may lead to inefficiencies in energy utilization. Tong et al. (55) found that *Prevotella* was significantly and positively correlated with propionic acid content. In the present study, the *Prevotellaceae_UCG-003* content was higher in the L-FCR group and was significantly and positively correlated with propionic acid content, which was increased to help reduce methane emissions. *Bacteroidaceae*, *Bacteroides*, and *Fusicatenibacter* were significantly and positively correlated with SCFA content. They were associated with cellulose and hemicellulose digestion, volatile fatty acid (VFA) production, and metabolism of the host. *Fusicatenibacter* and *Agathobacter* both belong to *Firmicutes*. In the present study, *Agathobacter* was significantly correlated with FCR, etc., whereas in the study of Gao et al. (56), it was found that *Agathobacter* may be associated with the synthesis of indole and its derivatives. Indole and its derivatives are known to be involved in host glucose and lipid metabolism, regulation of immune response, intestinal barrier function, and regulation of gut microbial community. *Prevotella*, as a beneficial gut bacterium, thrives in lower-pH environments and is commonly found in various dietary structures, participating in key processes such as polysaccharide and protein breakdowns, as well as sugar fermentation (57). Zhou et al. (58) found that *Prevotella* was significantly associated with host differentially expressed genes in the rumen and rectum of cattle from different RFI groups and that *Prevotella* modulated host traits related to feed efficiency. The results of the present study are similar to that study. *Prevotellaceae_UCG-003* efficiently metabolizes to fatty acids and belongs to the *Bacteroidota*, whose primary function is to degrade a wide range of plant polysaccharides, thereby enhancing nutrient utilization and host immunity (59). In conclusion, we hypothesized that gut microorganisms regulate SCFA production and content by interacting with SCFAs in the gastrointestinal tract, thereby affecting host digestive metabolism, organismal health, intestinal barrier function, and intestinal homeostasis. This process may in turn have an important impact on host feed conversion and fat deposition.

### Expression levels of nutrient transporters and digestive enzyme activity

In wild-type rats, the pH of intestinal epithelial cells decreased after the absorption of glycine-sarcosine dipeptide, but this change did not occur in *PEPT1*-deficient rats, suggesting that *PEPT1* functions as an acid transporter (60). *PEPT1* also regulates proton-dependent nutrient transport, and reduced expression of *PEPT1* leads to an increase in intracellular pH, which in turn may increase fat deposition (61). This result was also illustrated by the expression level of *PEPT1* in this study. *GLUT2* at the basement membrane assists in the transport of glucose and fructose into the interstitial fluid, thus providing more energy supply to the host, which suggests that the Hu sheep in the L-FCR group have more energy supply than the H-FCR group (62). *CAT1*, *rBAT*, and *B^0^AT* are responsible for the transport of neutral, essential, and psychoactive amino acids, which are important for maintaining amino acid balance and uptake as well as nerve signaling (63). *FATP4* is primarily located in cell membranes and facilitates fatty acid transport by binding and activating fatty acyl-coenzyme A (CoA). Inside the cell, fatty acids play a crucial role in energy metabolism and lipid synthesis. *FATP4* assists in the transport of fatty acids across cell membranes into the cytoplasm for energy production or lipid synthesis by converting them into fatty acyl-CoA (64). Intestinal digestive enzyme activities vary with diet type and also reflect the effect of diet on feed efficiency and growth performance of the host (65). The jejunum plays an important biological role as a key site for the digestion and absorption of nutrients in the host (66). Although there was no significant difference in the digestive enzyme activities in the jejunum of Hu sheep between the H-FCR and L-FCR groups, the digestive enzyme activities in the L-FCR group were higher than those in the high FCR group. Katarzyna et al. showed that the expression levels of intestinal nutrient transporter proteins were significantly higher in fast-growing Ross chickens than in slow-growing Athens Canadian Randombred Control (ACRBC) chickens, suggesting that higher levels of intestinal nutrient transport were associated with higher growth and feed efficiency of the host. The results of our study were similar to this study (67). In summary, we hypothesized that the L-FCR group may improve digestion and absorption of nutrients by upregulating genes encoding intestinal nutrient transporter carriers and digestive enzyme activities in Hu sheep. Notably, the expression level of fatty acid transporters in the jejunum showed the most significant increase, indicating that the jejunum plays a key role in lipid metabolism in the body.

### Conclusion

FCR was significantly correlated with production performance in Hu sheep. L-FCR Hu sheep were able to increase digestion and absorption of nutrients by increasing the activity of digestive enzymes and the number of nutrient transporter carriers in the small intestines, thus showing better production performance (higher ADG, better feed efficiency, and lower fat content in meat). The content of SCFA in the jejunum was significantly correlated with fat deposition traits, and there was a significant positive correlation between the relative abundance of *Aeromonas*, *Bacteroides*, *Fusicatenibacter*, and *Prevotellace_UCG_003* with jejunal SCFA content. As to whether the number and height of intestinal villi and biological pathways in the fore-digestive tract change when FCR is used as a selection index for Hu sheep, further research is needed to demonstrate this.

## Data Availability

The sequence files in this study were deposited at the Sequence Read Archive (SRA; https://www.ncbi.nlm.nih.gov/sra/PRJNA1019645).

## SUPPLEMENTAL MATERIAL

Supplemental figures and tables spectrum.01423-24-s0001.pdf Figures S1 and S2, and Table S1 to S4.

ASM does not own the copyrights to Supplemental Material that may be linked to, or accessed through, an article. The authors have granted ASM a non-exclusive, world-wide license to publish the Supplemental Material files. Please contact the corresponding author directly for reuse.

## References

[1] Groeneveld LF, Lenstra JA, Eding H, Toro MA, Scherf B, Pilling D, Negrini R, Finlay EK, Jianlin H, Groeneveld E, Weigend S, GLOBALDIV Consortium. 2010. Genetic diversity in farm animals – a review. Anim Genet 41:6–31. doi:10.1111/j.1365-2052.2010.02038.x 20500753

[2] Eydivandi S, Roudbar MA, Karimi MO, Sahana G. 2021. Genomic scans for selective sweeps through haplotype homozygosity and allelic fixation in 14 indigenous sheep breeds from Middle East and South Asia. Sci Rep 11:2834. doi:10.1038/s41598-021-82625-2 33531649 PMC7854752

[3] Yang Z, Yang X, Liu G, Deng M, Sun B, Guo Y, Liu D, Li Y. 2022. Polymorphisms in BMPR-IB gene and their association with litter size trait in Chinese Hu sheep. Anim Biotechnol 33:250–259. doi:10.1080/10495398.2020.1789158 32657205

[4] Montossi F, Font-i-Furnols M, del Campo M, San Julián R, Brito G, Sañudo C. 2013. Sustainable sheep production and consumer preference trends: compatibilities, contradictions, and unresolved dilemmas. Meat Sci 95:772–789. doi:10.1016/j.meatsci.2013.04.048 23769133

[5] Marzocchi MZ, Sakamoto LS, Canesin RC, Dos Santos Gonçalves Cyrillo J, Mercadante MEZ. 2020. Evaluation of test duration for feed efficiency in growing beef cattle. Trop Anim Health Prod 52:1533–1539. doi:10.1007/s11250-019-02161-0 31813088

[6] Cantalapiedra-Hijar G, Abo-Ismail M, Carstens GE, Guan LL, Hegarty R, Kenny DA, McGee M, Plastow G, Relling A, Ortigues-Marty I. 2018. Review: biological determinants of between-animal variation in feed efficiency of growing beef cattle. Animal 12:s321–s335. doi:10.1017/S1751731118001489 30139392

[7] Zhang X, Li G, Li F, Zhang D, Yuan L, Zhao Y, Zhang Y, Li X, Song Q, Wang W. 2023. Effect of feed efficiency on growth performance, body composition, and fat deposition in growing Hu lambs. Anim Biotechnol 34:183–198. doi:10.1080/10495398.2021.1951747 34346280

[8] Richardson EC, Herd RM, Archer JA, Woodgate RT, Arthur PF. 1998. Steers bred for improved net feed efficiency eat less for the same feedlot performance. Anim Prod Aus 22:213–216. https://api.semanticscholar.org/CorpusID:55146189.

[9] Zhang X, Wang W, Mo F, La Y, Li C, Li F. 2017. Association of residual feed intake with growth and slaughtering performance, blood metabolism, and body composition in growing lambs. Sci Rep 7:12681. doi:10.1038/s41598-017-13042-7 28978940 PMC5627304

[10] Nicholson JK, Holmes E, Kinross J, Burcelin R, Gibson G, Jia W, Pettersson S. 2012. Host-gut microbiota metabolic interactions. Science 336:1262–1267. doi:10.1126/science.1223813 22674330

[11] Montanholi Y, Fontoura A, Swanson K, Coomber B, Yamashiro S, Miller S. 2013. Small intestine histomorphometry of beef cattle with divergent feed efficiency. Acta Vet Scand 55:9. doi:10.1186/1751-0147-55-9 23379622 PMC3598877

[12] Sekirov I, Russell SL, Antunes LCM, Finlay BB. 2010. Gut microbiota in health and disease. Physiol Rev 90:859–904. doi:10.1152/physrev.00045.2009 20664075

[13] Giger-Reverdin S, Domange C, Broudiscou LP, Sauvant D, Berthelot V. 2020. Rumen function in goats, an example of adaptive capacity. J Dairy Res 87:45–51. doi:10.1017/S0022029920000060 33213566

[14] Cheng L, Cantalapiedra-Hijar G, Meale SJ, Rugoho I, Jonker A, Khan MA, Al-Marashdeh O, Dewhurst RJ. 2021. Review: markers and proxies to monitor ruminal function and feed efficiency in young ruminants. Animal 15:100337. doi:10.1016/j.animal.2021.100337 34537442

[15] Rook JA. 1964. Ruminal volatile fatty acid production in relation to animal production from grass. Proc Nutr Soc 23:71–80. doi:10.1079/pns19640013 14116006

[16] Johnson DE, Johnson KA, Baldwin RL. 1990. Changes in liver and gastrointestinal tract energy demands in response to physiological workload in ruminants. J Nutr 120:649–655. doi:10.1093/jn/120.6.649 2191096

[17] Bergamaschi M, Tiezzi F, Howard J, Huang YJ, Gray KA, Schillebeeckx C, McNulty NP, Maltecca C. 2020. Gut microbiome composition differences among breeds impact feed efficiency in swine. Microbiome 8:110. doi:10.1186/s40168-020-00888-9 32698902 PMC7376719

[18] Tan Z, Yang T, Wang Y, Xing K, Zhang F, Zhao X, Ao H, Chen S, Liu J, Wang C. 2017. Metagenomic analysis of cecal microbiome identified microbiota and functional capacities associated with feed efficiency in landrace finishing pigs. Front Microbiol 8:1546. doi:10.3389/fmicb.2017.01546 28848539 PMC5554500

[19] Gäbel G, Sehested J. 1997. SCFA transport in the forestomach of ruminants. Comp Biochem Physiol A Physiol 118:367–374. doi:10.1016/s0300-9629(96)00321-0 9366072

[20] Watanabe DHM, Doelman J, Steele MA, Guan LL, Seymour DJ, Penner GB. 2023. A comparison of post-ruminal provision of Ca-gluconate and Ca-butyrate on growth performance, gastrointestinal barrier function, short-chain fatty acid absorption, intestinal histology, and brush-border enzyme activity in beef heifers. J Anim Sci 101:skad050. doi:10.1093/jas/skad050 36799118 PMC10022388

[21] Liu YR, Du HS, Wu ZZ, Wang C, Liu Q, Guo G, Huo WJ, Zhang YL, Pei CX, Zhang SL. 2020. Branched-chain volatile fatty acids and folic acid accelerated the growth of Holstein dairy calves by stimulating nutrient digestion and rumen metabolism. Animal 14:1176–1183. doi:10.1017/S1751731119002969 31840620

[22] Sillence MN. 2004. Technologies for the control of fat and lean deposition in livestock. Vet J 167:242–257. doi:10.1016/j.tvjl.2003.10.020 15080873

[23] Horodyska J, Hamill RM, Varley PF, Reyer H, Wimmers K. 2017. Genome-wide association analysis and functional annotation of positional candidate genes for feed conversion efficiency and growth rate in pigs. PLoS One 12:e0173482. doi:10.1371/journal.pone.0173482 28604785 PMC5467825

[24] Zhang Y, Zhang X, Li F, Li C, Zhang D, Li X, Zhao Y, Wang W. 2021. Exploring the ruminal microbial community associated with fat deposition in lambs. Animals (Basel) 11:3584. doi:10.3390/ani11123584 34944359 PMC8698113

[25] Wang Y, Heng C, Zhou X, Cao G, Jiang L, Wang J, Li K, Wang D, Zhan X. 2021. Supplemental Bacillus subtilis DSM 29784 and enzymes, alone or in combination, as alternatives for antibiotics to improve growth performance, digestive enzyme activity, anti-oxidative status, immune response and the intestinal barrier of broiler chickens. Br J Nutr 125:494–507. doi:10.1017/S0007114520002755 32693847 PMC7885174

[26] Magoč T, Salzberg SL. 2011. FLASH: fast length adjustment of short reads to improve genome assemblies. Bioinformatics 27:2957–2963. doi:10.1093/bioinformatics/btr507 21903629 PMC3198573

[27] Bokulich NA, Subramanian S, Faith JJ, Gevers D, Gordon JI, Knight R, Mills DA, Caporaso JG. 2013. Quality-filtering vastly improves diversity estimates from Illumina amplicon sequencing. Nat Methods 10:57–59. doi:10.1038/nmeth.2276 23202435 PMC3531572

[28] Edgar RC, Haas BJ, Clemente JC, Quince C, Knight R. 2011. UCHIME improves sensitivity and speed of chimera detection. Bioinformatics 27:2194–2200. doi:10.1093/bioinformatics/btr381 21700674 PMC3150044

[29] Haas BJ, Gevers D, Earl AM, Feldgarden M, Ward DV, Giannoukos G, Ciulla D, Tabbaa D, Highlander SK, Sodergren E, Methé B, DeSantis TZ, Human Microbiome Consortium, Petrosino JF, Knight R, Birren BW. 2011. Chimeric 16S rRNA sequence formation and detection in Sanger and 454-pyrosequenced PCR amplicons. Genome Res 21:494–504. doi:10.1101/gr.112730.110 21212162 PMC3044863

[30] Wang W, Cheng L, Guo J, Ma Y, Li F. 2014. Expression of Ghrelin in gastrointestinal tract and the effect of early weaning on Ghrelin expression in lambs. Mol Biol Rep 41:909–914. doi:10.1007/s11033-013-2935-2 24385298

[31] Livak KJ, Schmittgen TD. 2001. Analysis of relative gene expression data using real-time quantitative PCR and the 2^−ΔΔCT^ method. Methods 25:402–408. doi:10.1006/meth.2001.1262 11846609

[32] Basarab JA, Beauchemin KA, Baron VS, Ominski KH, Guan LL, Miller SP, Crowley JJ. 2013. Reducing GHG emissions through genetic improvement for feed efficiency: effects on economically important traits and enteric methane production. Animal 7:303–315. doi:10.1017/S1751731113000888 23739472 PMC3691002

[33] Hoque MA, Suzuki K, Kadowaki H, Shibata T, Oikawa T. 2007. Genetic parameters for feed efficiency traits and their relationships with growth and carcass traits in Duroc pigs. J Anim Breed Genet 124:108–116. doi:10.1111/j.1439-0388.2007.00650.x 17550351

[34] Wen C, Yan W, Zheng J, Ji C, Zhang D, Sun C, Yang N. 2018. Feed efficiency measures and their relationships with production and meat quality traits in slower growing broilers. Poult Sci 97:2356–2364. doi:10.3382/ps/pey062 29669019

[35] Detweiler RA, Pringle TD, Rekaya R, Wells JB, Segers JR. 2019. The impact of selection using residual average daily gain and marbling EPDs on growth, performance, and carcass traits in Angus steers1. J Anim Sci 97:2450–2459. doi:10.1093/jas/skz124 31100117 PMC6541813

[36] Zhao Y, Zhang X, Li F, Zhang D, Zhang Y, Li X, Song Q, Li C, Zhao L, Wang J, Xu D, Cheng J, Li W, Lin C, Zhou B, Wang W. 2023. Estimation of genetic correlations of two key feed efficiency traits with production traits in male Hu sheep. Anim Biotechnol 34:2805–2816. doi:10.1080/10495398.2022.2119405 36074803

[37] Gurgeira DN, Crisóstomo C, Sartori LVC, de Paz CCP, Delmilho G, Chay-Canul AJ, Bedoya HJN, Vega WHO, Bueno MS, da Costa RLD. 2022. Characteristics of growth, carcass and meat quality of sheep with different feed efficiency phenotypes. Meat Sci 194:108959. doi:10.1016/j.meatsci.2022.108959 36084489

[38] Zeng H, Yin Y, Chen L, Xu Z, Luo Y, Wang Q, Yang B, Wang J. 2023. Alterations in nutrient digestion and utilization associated with different residual feed intake in Hu sheep. Anim Nutr 13:334–341. doi:10.1016/j.aninu.2023.02.009 37207113 PMC10189385

[39] Rastogi S, Mohanty S, Sharma S, Tripathi P. 2022. Possible role of gut microbes and host’s immune response in gut-lung homeostasis. Front Immunol 13:954339. doi:10.3389/fimmu.2022.954339 36275735 PMC9581402

[40] Wu T, Wang G, Xiong Z, Xia Y, Song X, Zhang H, Wu Y, Ai L. 2022. Probiotics interact with lipids metabolism and affect gut health. Front Nutr 9:917043. doi:10.3389/fnut.2022.917043 35711544 PMC9195177

[41] Hernández MAG, Canfora EE, Jocken JWE, Blaak EE. 2019. The short-chain fatty acid acetate in body weight control and insulin sensitivity. Nutrients 11:1943. doi:10.3390/nu11081943 31426593 PMC6723943

[42] Zhang J, Shi H, Wang Y, Cao Z, Yang H, Li S. 2018. Effect of limit-fed diets with different forage to concentrate ratios on fecal bacterial and archaeal community composition in holstein heifers. Front Microbiol 9:976. doi:10.3389/fmicb.2018.00976 29867879 PMC5962747

[43] Aliakbari A, Zemb O, Cauquil L, Barilly C, Billon Y, Gilbert H. 2022. Microbiability and microbiome-wide association analyses of feed efficiency and performance traits in pigs. Genet Sel Evol 54:29. doi:10.1186/s12711-022-00717-7 35468740 PMC9036775

[44] Wang X, Zhang Z, Wang X, Bao Q, Wang R, Duan Z. 2021. The impact of host genotype, intestinal sites and probiotics supplementation on the gut microbiota composition and diversity in sheep. Biology (Basel) 10:769. doi:10.3390/biology10080769 34440001 PMC8389637

[45] Tan Z, Wang Y, Yang T, Ao H, Chen S, Xing K, Zhang F, Zhao X, Liu J, Wang C. 2018. Differences in gut microbiota composition in finishing Landrace pigs with low and high feed conversion ratios. Antonie Van Leeuwenhoek 111:1673–1685. doi:10.1007/s10482-018-1057-1 29497869 PMC6097733

[46] Zeng H, Guo C, Sun D, Seddik HE, Mao S. 2019. The ruminal microbiome and metabolome alterations associated with diet-induced milk fat depression in dairy cows. Metabolites 9:154. doi:10.3390/metabo9070154 31340604 PMC6680951

[47] Conte G, Dimauro C, Daghio M, Serra A, Mannelli F, McAmmond BM, Van Hamme JD, Buccioni A, Viti C, Mantino A, Mele M. 2022. Exploring the relationship between bacterial genera and lipid metabolism in bovine rumen. Animal 16:100520. doi:10.1016/j.animal.2022.100520 35468508

[48] Lopez-Zavala AA, Guevara-Hernandez E, Vazquez-Lujan LH, Sanchez-Paz A, Garcia-Orozco KD, Contreras-Vergara CA, Lopez-Leal G, Arvizu-Flores AA, Ochoa-Leyva A, Sotelo-Mundo RR. 2018. A novel thymidylate synthase from the Vibrionales, Alteromonadales, Aeromonadales, and Pasteurellales (VAAP) clade with altered nucleotide and folate binding sites. PeerJ 6:e5023. doi:10.7717/peerj.5023 29922516 PMC6005164

[49] Prochazkova P, Roubalova R, Dvorak J, Kreisinger J, Hill M, Tlaskalova-Hogenova H, Tomasova P, Pelantova H, Cermakova M, Kuzma M, Bulant J, Bilej M, Smitka K, Lambertova A, Holanova P, Papezova H. 2021. The intestinal microbiota and metabolites in patients with anorexia nervosa. Gut Microbes 13:1–25. doi:10.1080/19490976.2021.1902771 PMC8018350 33779487

[50] Guo X, Sha Y, Lv W, Pu X, Liu X, Luo Y, Hu J, Wang J, Li S, Zhao Z. 2022. Sex differences in rumen fermentation and microbiota of Tibetan goat. Microb Cell Fact 21:55. doi:10.1186/s12934-022-01783-8 35392919 PMC8991483

[51] Teobaldo RW, Granja-Salcedo YT, Cardoso A da S, Constancio MTL, Brito TR, Romanzini EP, Reis RA. 2023. The impact of mineral and energy supplementation and phytogenic compounds on rumen microbial diversity and nitrogen utilization in grazing beef cattle. Microorganisms 11:810. doi:10.3390/microorganisms11030810 36985382 PMC10051884

[52] Liu Y, Yang J, Liu X, Liu R, Wang Y, Huang X, Li Y, Liu R, Yang X. 2023. Dietary folic acid addition reduces abdominal fat deposition mediated by alterations in gut microbiota and SCFA production in broilers. Anim Nutr 12:54–62. doi:10.1016/j.aninu.2022.08.013 36439290 PMC9684696

[53] Baird GD, Lomax MA, Symonds HW, Shaw SR. 1980. Net hepatic and splanchnic metabolism of lactate, pyruvate and propionate in dairy cows in vivo in relation to lactation and nutrient supply. Biochem J 186:47–57. doi:10.1042/bj1860047 6989361 PMC1161502

[54] Carmichael MN, Dycus MM, Lourenco JM, Welch CB, Davis DB, Krause TR, Rothrock MJ, Fluharty FL, Pringle TD, Callaway TR. 2024. Ruminal microbiome differences in angus steers with differing feed efficiencies during the feedlot finishing phase. Microorganisms 12:536. doi:10.3390/microorganisms12030536 38543587 PMC10972478

[55] Tong J, Zhang H, Wang J, Liu Y, Mao S, Xiong B, Jiang L. 2020. Effects of different molecular weights of chitosan on methane production and bacterial community structure in vitro. J Integr Agric 19:1644–1655. doi:10.1016/S2095-3119(20)63174-4

[56] Gao M, Wu J, Zhou S, Chen Y, Wang M, He W, Jiang L, Shu Y, Wang X. 2024. Combining fecal microbiome and metabolomics reveals diagnostic biomarkers for esophageal squamous cell carcinoma. Microbiol Spectr 12:e0401223. doi:10.1128/spectrum.04012-23 38497715 PMC11064534

[57] Bach A, López-García A, González-Recio O, Elcoso G, Fàbregas F, Chaucheyras-Durand F, Castex M. 2019. Changes in the rumen and colon microbiota and effects of live yeast dietary supplementation during the transition from the dry period to lactation of dairy cows. J Dairy Sci 102:6180–6198. doi:10.3168/jds.2018-16105 31056321

[58] Zhou X, Ma Y, Yang C, Zhao Z, Ding Y, Zhang Y, Wang P, Zhao L, Li C, Su Z, Wang X, Ming W, Zeng L, Kang X. 2023. Rumen and fecal microbiota characteristics of qinchuan cattle with divergent residual feed intake. Microorganisms 11:358. doi:10.3390/microorganisms11020358 36838323 PMC9964965

[59] Bayliss CE, Houston AP. 1984. Characterization of plant polysaccharide- and mucin-fermenting anaerobic bacteria from human feces. Appl Environ Microbiol 48:626–632. doi:10.1128/aem.48.3.626-632.1984 6093693 PMC241577

[60] Chen M, Singh A, Xiao F, Dringenberg U, Wang J, Engelhardt R, Yeruva S, Rubio-Aliaga I, Nässl A-M, Kottra G, Daniel H, Seidler U. 2010. Gene ablation for PEPT1 in mice abolishes the effects of dipeptides on small intestinal fluid absorption, short-circuit current, and intracellular pH. Am J Physiol Gastrointest Liver Physiol 299:G265–G274. doi:10.1152/ajpgi.00055.2010 20430876

[61] Brooks KK, Liang B, Watts JL. 2009. The influence of bacterial diet on fat storage in C. elegans. PLoS One 4:e7545. doi:10.1371/journal.pone.0007545 19844570 PMC2760100

[62] Thorens B, Charron MJ, Lodish HF. 1990. Molecular physiology of glucose transporters. Diabetes Care 13:209–218. doi:10.2337/diacare.13.3.209 2407476

[63] Bröer S. 2008. Amino acid transport across mammalian intestinal and renal epithelia. Physiol Rev 88:249–286. doi:10.1152/physrev.00018.2006 18195088

[64] Ibrahim A, Yucel N, Kim B, Arany Z. 2020. Local mitochondrial ATP production regulates endothelial fatty acid uptake and transport. Cell Metab 32:309–319. doi:10.1016/j.cmet.2020.05.018 32521232 PMC7415739

[65] Zhang H, Ding Q, Wang A, Liu Y, Teame T, Ran C, Yang Y, He S, Zhou W, Olsen RE, Zhang Z, Zhou Z. 2020. Effects of dietary sodium acetate on food intake, weight gain, intestinal digestive enzyme activities, energy metabolism and gut microbiota in cultured fish: zebrafish as a model. Aquaculture 523:735188. doi:10.1016/j.aquaculture.2020.735188

[66] Zhu HL, Zhao XW, Han RW, DU QJ, Qi YX, Jiang HN, Huang DW, Yang YX. 2021. Changes in bacterial community and expression of genes involved in intestinal innate immunity in the jejunum of newborn lambs during the first 24 hours of life. J Dairy Sci 104:9263–9275. doi:10.3168/jds.2020-19888 33985780

[67] Miska KB, Fetterer RH. 2019. Expression of amino acid and sugar transporters, aminopeptidase, and the di- and tri-peptide transporter PepT1; differences between modern fast growing broilers and broilers not selected for rapid growth. Poult Sci 98:2272–2280. doi:10.3382/ps/pey583 30624759

