# Supplementary material for: Differences in production performance, fore-digestive tract microbiota, and expression levels of nutrient transporters of Hu sheep with different feed conversion ratio
Source: Microbiol Spectr. 2025 Apr 17;13(6):e01423-24. doi: 10.1128/spectrum.01423-24 (PMC12131840; doi:10.1128/spectrum.01423-24)
Supplement: Supplemental figures and tables — Figures S1 and S2, and Table S1 to S4. [file spectrum.01423-24-s0001.pdf]

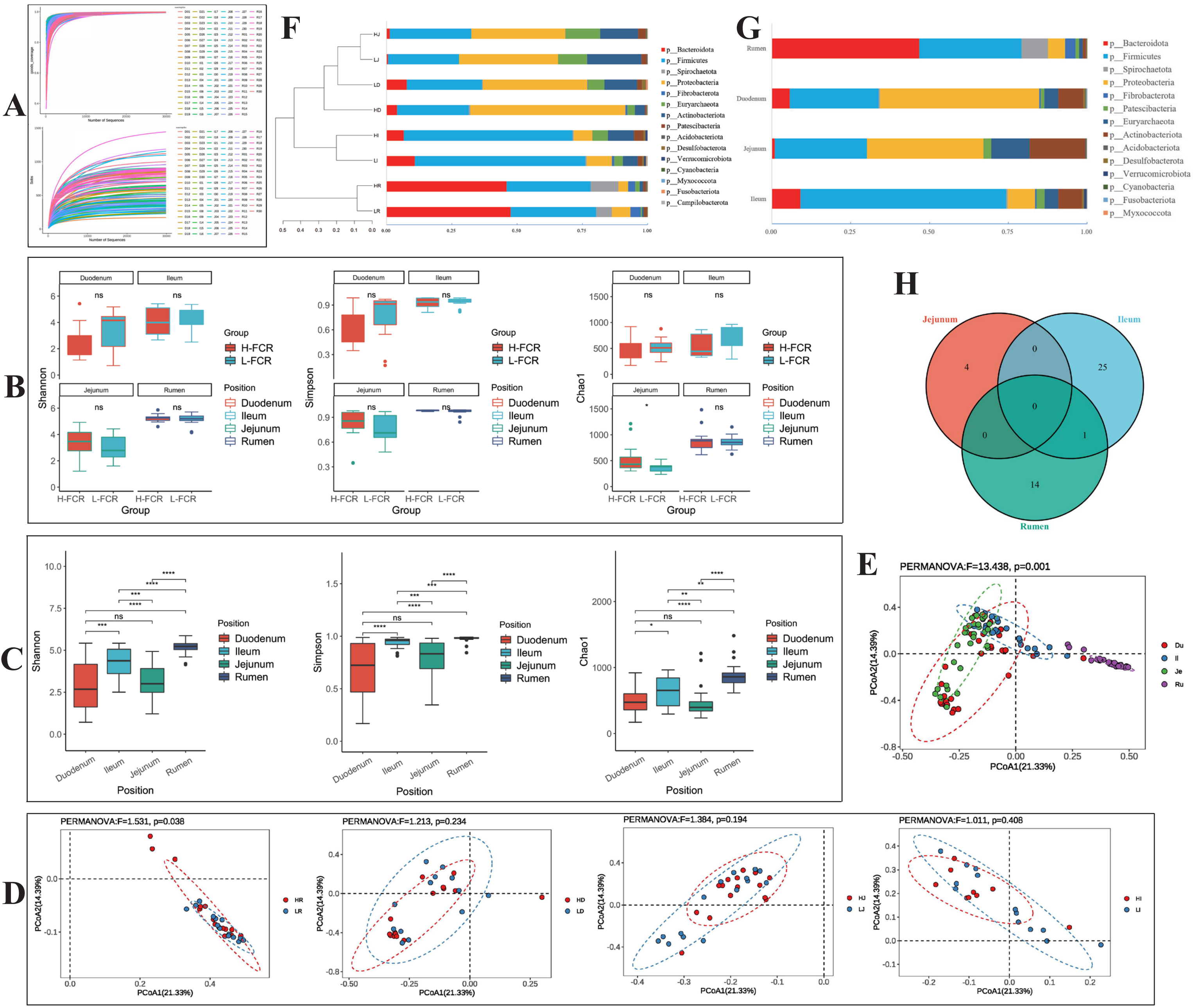

Supplementary Figure S1

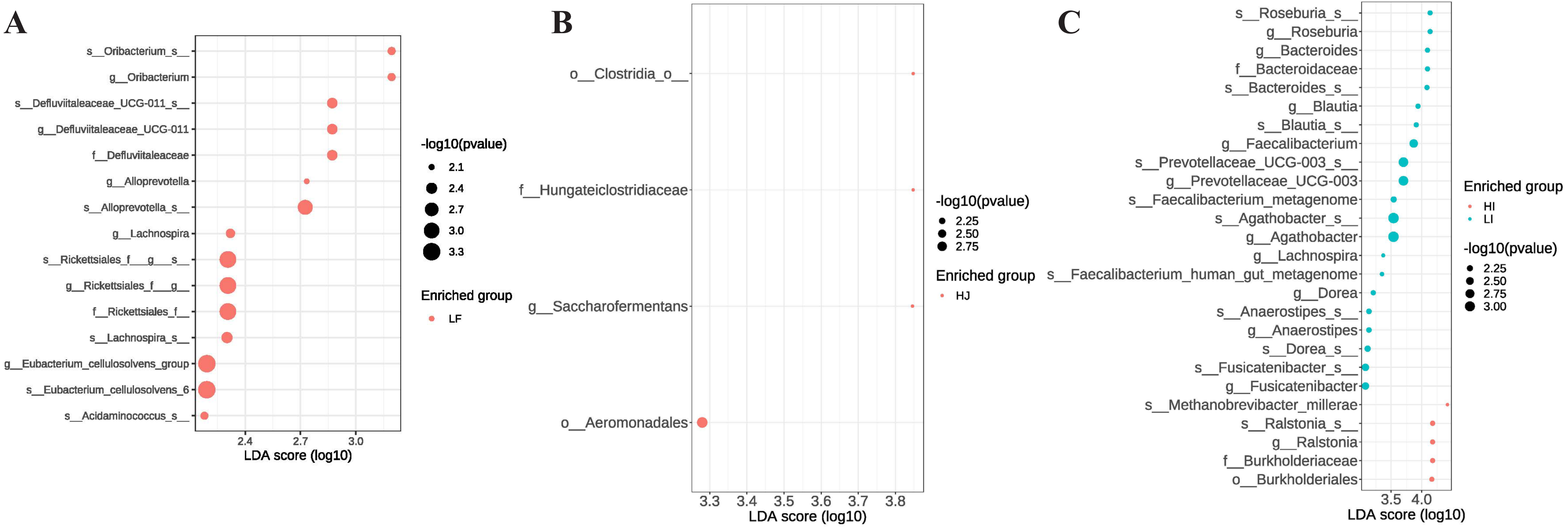

Supplementary Figure S2

Supplemental Figures

Supplementary Figure S1. Comparison of microbial diversity and composition in rumen and small intestine. A. Rumen and small intestine observed features curve. In the figure, D denotes duodenum, J denotes jejunum, I denotes ileum, and R denotes rumen. B. Comparison of Alpha diversity in rumen and small intestine between H-FCR and L-FCR groups. * indicates a significant difference ($P < 0.05$), and ns shows no significant difference ($P > 0.05$). H and L denote high and low FCR groups, and D, J, I, and R denote duodenum, jejunum, ileum, and rumen, respectively. C. Comparison of Alpha diversity in rumen and small intestine. * or ** or *** or **** indicates a significant difference ($P < 0.05$), and ns shows no significant difference ($P > 0.05$). D. Comparison of Beta diversity in rumen and small intestine between H-FCR and L-FCR groups. H and L denote high and low FCR groups, and D, J, I, and R denote duodenum, jejunum, ileum, and rumen, respectively. E. Comparison of Beta diversity in rumen and small intestine. F. Species composition of rumen and small intestine in H-FCR and L-FCR groups. G. Species composition of rumen and small intestine. H. Venn diagram of differential microbiota in the rumen, jejunum, and ileum.

Supplementary Figure S2. LEfSe analysis of different groups. (A. Rumen, B. Jejunum, C. Ileum). L and H represent L-FCR and H-FCR groups, respectively. LF represents the rumen of the L-FCR group, while HJ represents the ileum of the H-FCR group. HI and LI refer to the ileum of the H-FCR and L-FCR group, respectively.

Supplemental Tables

Supplementary Table S1. Feed Formulation and Nutrient Levels.

Ingredients	Percentage (%)	Chemical composition	Content
Corn	32.50	Dry matter (DM) (%)	88.78
Corn germ meal	18.00	Crude protein (CP) (%)	13.09
Corn stalks	12.00	Digestible energy (MJ/kg)	11.11
Corn hulls	11.00	Crude fiber (CF) (%)	9.78
Corn cob	8.00	Ether extract (EE) (%)	1.72
Soybean meal	4.00	Acid Detergent Lignin (ADL) (%)	21.15
Cotton meal	5.00	Nitrogen free extract (NFE) (%)	55.05
Molasses	3.30	Neutral detergent fiber (NDF) (%)	27.08
Bentonite	1.50	Acid detergent fiber (ADF) (%)	13.99
Baking soda	1.00		
Stone powder	0.80		
Expanded Urea	0.70		
NaCl	0.70		
Gypsum powder	1.00		
Premix	0.50		
Total	100.00		

The premix included the following per kg of the starter diet: 25 mg Fe as $FeSO_4 \cdot H_2O$; 40 mg Zn as $ZnSO_4 \cdot H_2O$; 8 mg Cu as $CuSO_4 \cdot 5H_2O$; 40 mg Mn as $MnSO_4 \cdot H_2O$; 0.3 mg I as KI; 0.2 mg Se as Na_2SeO_3; 0.1 mg Co as $CoCl_2$; 940 IU vitamin A; 111 IU vitamin D; 20 IU vitamin E; and 0.02 mg vitamin B_{12}.

Supplementary Table S2. Small intestine tissue expresses gene primers.

Gene	Primer	Primer sequence (5'-3')	GenBank accession number	Size (bp)
SGLT1	*SGLT1*-F	TGCACTACTTGTACTTTGCCAT	NM_001009404	179
	SGLT1-R	GGCATCTTGAATGTCCTCGTCT		
GLUT2	*GLUT2*-F	CTTTAGCAATAGCTGCGTTC	XM_004003162	131
	GLUT2-R	TAGAGTAAAGGCCAAGACCAC		
GLUT5	*GLUT5*-F	GTAAGACCAGCTCCTATCACA	NM_001009451	132
	GLUT5-R	TTCCACTCTGAACACATGCCT		
PEPT1	*PEPT1*-F	GCCTGAACTCCTTTTCCTAACAC	NM_001009758	186
	PEPT1-R	AGTTAATACCAGCCGGTCCT		
CAT1	*CAT1*-F	AGATAAGTTTCCTGCTTACGTT	XM_027973630	168
	CAT1-R	ACACAATCCTCTTAATAATGGCT		
rBAT	*rBAT*-F	ACCGGACAGGAAAATATACTGA	XM_042246231	122
	rBAT-R	CTCATCAAAGTGCCAACTGGA		
B^0AT	B^0AT-F	TCGCGTCCATGTTCGTGTCCT	XM_027980192	157
	B^0AT-R	CGGGCACACTCCTCCACGTA		
EAAT3	*EAAT3*-F	CTCCAGCCAATTTCAAACGAA	XM_004004350	146
	EAAT3-R	GTCAGTAGTTACCAGCGTCC		
FATP4	*FATP4*-F	GCTGAAACTGCCCTGGACCCAA	XM_015094163	116
	FATP4-R	CGCCGAAGATATCACGCCTGACA		
GAPDH	*GAPDH*-F	AGATGGTGAAGGTCGGAGTG	XM_027961471	188
	GAPDH-R	GTTCTCTGCCTTGACTGTGC		

Supplementary Table S3. Sequencing data production statistics.

Sample Name	Raw PE(#)	Raw Tags(#)	Clean Tags(#)	Effective Tags	Q20	Q30	GC%
D01	79,528	78,036	77,399	68,513	98.28	94.33	52.41
D02	69,627	56,240	55,672	52,906	98.05	93.7	50.11
D03	84,374	82,168	80,321	74,954	97.17	91.94	51.03
D04	88,864	86,458	85,702	77,501	98.13	93.88	51.03
D05	76,930	76,551	75,254	67,674	97.59	92.77	51.69
D06	88,794	68,453	67,767	64,373	97.76	92.9	50.91
D07	90,423	75,992	75,188	70,417	97.96	93.51	51.07
D08	76,876	63,089	62,489	56,157	98.38	94.52	53.45
D09	94,846	94,208	93,457	73,638	98.54	95.04	53.44
D10	67,969	58,666	58,203	48,997	98.57	95.11	51.87
D11	81,013	78,365	78,044	61,590	98.18	94.25	54.28
D12	85,372	78,663	78,048	69,868	98.7	95.37	53.49
D13	92,385	91,140	90,260	81,568	98.05	93.7	51.13
D14	89,330	87,575	86,390	74,192	97.44	92.38	48.33
D15	90,718	78,131	77,526	65,724	98.48	94.92	54.27
D16	62,005	52,216	51,246	46,325	97.4	92.34	51.97
D17	64,877	63,635	63,261	44,704	98.1	93.89	52.4
D18	61,616	56,585	56,174	48,616	98.56	95.13	53.5
D19	82,690	82,285	81,885	63,277	98.11	94.05	54.87
D21	79,242	77,285	76,453	67,694	98.04	93.69	51.33
D22	76,980	72,035	71,484	55,339	98.51	95.12	55.49
D23	86,662	86,592	85,953	65,889	98.48	94.91	53.3
D24	88,470	87,725	86,857	82,642	98.03	93.57	50.56
D26	89,555	89,340	88,702	71,550	98.48	94.93	53.04
D27	63,483	56,118	55,716	50,481	98.32	94.47	52.46
D28	78,081	71,304	70,800	60,203	98.67	95.39	53.63
D29	76,586	75,615	74,865	71,630	98.03	93.59	50.62
D30	60,267	58,175	57,792	45,962	98.55	95.19	54.47
I01	87,733	85,871	85,254	66,954	98.58	95.24	52.9
I02	87,541	84,638	84,106	67,542	98.46	94.88	52.71
I03	73,255	67,294	66,869	53,421	98.69	95.48	52.21
I04	85,060	82,119	81,751	66,629	98.46	94.99	53.76
I05	60,527	50,356	50,015	41,369	98.66	95.42	53.22
I08	86,574	85,197	84,748	68,375	98.49	95.01	54.76
I09	89,666	89,430	88,824	78,408	98.45	94.76	54.73
I14	78,637	77,434	76,888	62,646	98.18	94.2	53.65
I15	60,176	57,824	57,550	47,101	98.47	94.91	53.42
I16	77,382	73,248	72,678	54,869	98.54	95.03	53.78
I17	82,168	79,174	78,672	65,651	98.47	94.78	50.24
I18	77,967	77,650	77,170	61,768	98.53	95.03	53.42
I21	78,977	77,456	76,983	58,134	98.47	94.93	54.19

I22	88,069	86,540	86,083	67,651	98.29	94.42	54.61
I23	85,712	85,554	85,137	66,091	98.62	95.33	53.6
I24	85,658	83,259	82,697	63,158	98.57	95.04	53.31
I25	76,090	75,943	75,391	55,386	98.47	94.9	53.96
I26	54,358	54,267	53,923	42,536	98.56	95.2	53.27
I27	70,431	65,370	64,601	50,593	98.06	93.69	48.6
I28	92,283	89,395	88,857	67,747	98.59	95.2	53.74
I29	55,979	54,605	54,272	38,629	98.54	95.08	53.14
I30	63,631	63,064	62,766	49,846	98.53	95.06	52.63
J01	92,151	89,496	88,950	69,830	98.54	95.13	53.99
J02	64,217	61,521	61,237	51,125	98.45	94.81	53.57
J03	84,356	83,126	82,764	64,399	98.5	95	53.91
J04	64,574	58,619	58,245	47,397	98.38	94.7	53.42
J05	80,636	67,988	67,432	60,618	98.54	95.09	53.48
J06	80,357	77,991	77,462	69,192	98.5	94.98	53.96
J07	83,207	75,972	75,356	63,042	98.5	94.97	53.49
J08	86,997	67,681	67,202	62,717	98.43	94.7	52.48
J09	86,528	86,247	85,698	73,054	98.39	94.69	55.6
J10	83,965	81,845	81,227	72,712	98.35	94.64	55.67
J11	83,754	81,215	80,521	69,338	98.3	94.42	53.04
J12	88,984	86,265	84,434	70,643	96.96	91.53	52.79
J13	79,086	78,852	78,126	71,937	98.58	95.1	53.68
J14	80,390	77,490	74,645	70,814	96.37	90.25	51.06
J15	78,402	76,737	76,227	61,699	97.76	93.23	54.51
J16	86,568	71,279	70,608	66,928	98.15	93.95	53.49
J17	87,935	87,932	87,028	80,086	98	93.58	51.09
J18	78,148	77,207	76,403	69,610	97.87	93.3	51.43
J19	82,089	71,997	71,424	65,595	98.53	95.01	54.14
J20	95,468	93,529	92,923	76,609	98.58	95.22	55.17
J21	84,817	79,294	78,762	68,554	98.41	94.76	53.52
J22	87,029	84,927	84,343	63,522	98.12	94.05	56.73
J23	90,844	88,576	88,025	70,318	98.59	95.24	53.74
J24	83,730	75,896	75,235	68,052	98.02	93.62	51.57
J25	86,916	78,915	77,946	66,507	97.3	92.21	52.15
J26	93,835	91,399	90,774	73,623	98.43	94.78	54.62
J27	91,399	91,380	90,750	75,234	98.19	94.21	56.02
J28	81,231	78,319	77,821	63,209	98.5	95.02	55.06
J29	90,665	90,191	89,731	67,589	98.49	94.96	55.44
J30	87,086	84,105	81,618	69,210	96.39	90.45	51.71
R01	87,435	87,109	86,498	65,314	98.34	94.56	53.8
R02	84,863	83,293	82,680	65,246	98.22	94.25	53.49
R03	62,165	60,739	60,377	47,117	98.1	93.89	53.5
R04	78,244	72,075	71,704	55,233	98.59	95.23	53.26
R05	69,982	69,345	69,070	53,520	98.52	95.09	53.95

R06	91,125	89,524	89,050	68,010	98.55	95.01	53.56
R07	84,614	82,204	81,713	64,833	98.44	94.82	53.43
R08	68,267	66,477	66,279	53,425	98.33	94.6	53.52
R09	93,765	92,983	92,113	67,762	98.26	94.41	52.88
R10	89,846	88,725	87,963	67,272	98.34	94.57	53.11
R11	70,531	68,033	67,755	52,516	98.1	93.97	53.79
R12	82,245	80,398	80,096	65,518	97.55	92.45	53.93
R13	69,996	69,701	69,424	51,168	98.22	94.22	53.97
R14	80,637	79,228	78,833	64,393	98.44	94.85	54.31
R15	64,357	62,089	61,881	48,494	97.94	93.83	54.6
R16	71,078	68,934	68,662	55,209	98.39	94.74	54.77
R17	87,111	86,590	86,107	77,792	98.4	94.7	53.08
R18	57,128	55,218	54,895	40,645	98.5	95.03	53.5
R19	89,335	87,096	86,628	79,249	98.64	95.36	54.46
R20	82,071	71,929	71,540	62,549	98.55	95.13	53.74
R21	83,828	83,713	83,304	60,374	98.5	95.04	53.85
R22	76,733	74,361	73,668	58,703	98.26	94.49	53.51
R23	59,634	58,515	58,383	47,509	98.5	95.04	53.48
R24	87,086	85,949	85,528	63,970	98.48	94.95	53.78
R25	77,648	77,254	76,911	61,054	98.48	94.92	53.59
R26	77,655	75,902	75,367	57,323	98.09	93.99	53.63
R27	87,472	84,866	84,335	63,745	98.55	95.11	53.11
R28	82,887	80,127	79,699	62,514	98.47	94.96	53.24
R29	86,393	85,845	85,481	61,335	98.54	95	53.15
R30	82,221	80,750	80,260	64,107	98.27	94.35	53.35

Supplementary Table S4. Biochemical indices of blood in Hu sheep.

Items	H-FCR	L-FCR	*P*-Value
No.		30	
Alanine aminotransferase (ALT), U/L	5.53±0.97	7.94±0.78	0.063
Aspartate aminotransferase (AST), U/L	68.40±4.79	78.38±5.13	0.168
Total bilirubin (TBIL), μmol/L	0.56±0.11	0.56±0.09	0.997
Direct bilirubin (DBIL), μmol/L	0.87±0.10	0.85±0.10	0.926
Total protein (TP), g/L	54.07±3.43	55.46±2.42	0.742
Albumin (ALB), g/L	20.00±1.20	20.43±1.01	0.788
Alkaline phosphatase (ALP), U/L	197.50±26.44	223.20±21.51	0.455
Creatinine (CR), μmol/L	30.97±2.57	30.82±1.43	0.957
Triglyceride (TG), mmol/L	0.15±0.01	0.14±0.01	0.483
Lactate dehydrogenase (LDH), U/L	418.00±34.06	418.26±40.09	0.996
Creatine kinase (CK), U/L	170.85±21.38	157.99±14.96	0.622
Glucose (GLU), mmol/L	8.80±0.45	8.61±0.39	0.757

Note: The t-test was used to compare the differences between the two groups with 95% confidence intervals. Statistical data are expressed as mean ± standard deviation. Differences were considered significant when $P < 0.05$, highly significant when $P < 0.01$, and not significant when $P > 0.05$.
